# Covalent Organic Frameworks for Piezocatalysis, Piezo-Photocatalysis and Piezoelectric Energy Harvesting

**DOI:** 10.3390/nano16211350

**Published:** 2026-10-24

**Authors:** Kailai Zhang, Ao Feng, Gan Wang, Hang Chen, Shurui Xu, Guoyu Zhong, Baizeng Fang

**Affiliations:** 1 School of Chemistry and Chemical Engineering, South China University of Technology, Guangzhou 510641, China; zhangkailai@dgut.edu.cn; 2 Guangdong Provincial Key Laboratory of Multi-Energy Complementary Distributed Energy Systems, School of Chemical Engineering and Energy Technology, Dongguan University of Technology, Dongguan 523808, China; fengao514@163.com (A.F.); wanggan0103@163.com (G.W.); chenhang@dgut.edu.cn (H.C.); 3 Innovation Catalysis Team, School of Chemical Engineering and Energy Technology, Dongguan University of Technology, Dongguan 523808, China

**Keywords:** covalent organic frameworks, piezocatalysis, piezo-photocatalysis, piezoelectric energy harvesting, polarization engineering

## Abstract

Low-grade mechanical energy widely exists in natural environments, such as ocean-wave fluctuation, industrial vibration and human-body motion, yet most of this energy remains underutilized. Piezocatalysis, piezo-photocatalysis and piezoelectric nanogenerators (PENGs) provide feasible routes to convert ambient mechanical-vibration energy into chemical energy and usable electricity. Distinct from conventional inorganic piezoelectric materials suffering from low specific surface area and limited structural tunability, covalent organic frameworks (COFs) possess tailorable skeletons, high porosity, excellent chemical–thermal stability and modular symmetry-engineering capability. Non-centrosymmetric COFs with intrinsic piezoelectric responses can be realized via rational monomer-level design. Nevertheless, research on piezoelectric-related COFs-based systems is still in its early exploratory stage, and comprehensive reviews covering piezocatalysis, piezo-photocatalysis and piezoelectric energy harvesting remain scarce. This review addresses both intrinsic piezoelectric COFs and COFs-containing hybrid composite systems. Notably, performance gains in composite systems cannot be simply attributed to the intrinsic piezoelectric response of the COFs component. We systematically discuss their fundamental mechanisms, unique flexible-pore electromechanical features, multi-scale modification strategies, diverse application scenarios, and unresolved challenges. We discuss the applicability and inherent limitations of core piezocatalytic interpretive frameworks for flexible porous COFs systems. Five mainstream strategies for boosting COF piezoresponse are summarized: building-block molecular design, defect engineering, morphology modulation, single-atom metal anchoring and heterostructure construction. Representative applications including water-energy conversion, CO_2_ reduction, organic-pollutant remediation, uranium (VI) reduction, antibacterial disinfection and mechanical-energy harvesting are presented. Key bottlenecks covering mechanistic ambiguity, characterization constraints, synthetic hurdles and long-term practical stability are highlighted, and targeted future research directions are proposed. This review offers instructive guidelines for developing high-performance symmetry-engineered COFs-based piezofunctional systems.

## 1. Introduction

Global energy shortages and increasing environmental pollution have continuously driven the exploration of renewable-energy conversion technologies [1,2,3,4]. Photocatalysis has received tremendous research attention over recent decades [5,6,7,8]. Nevertheless, its practical deployment is severely restricted by the intermittency of solar illumination. By contrast, abundant low-grade mechanical energy ubiquitously occurs in nature and human activities, including ocean-wave oscillation, fluid turbulence, factory-equipment vibration, vehicle motion, and human physiological movement, and is difficult to collect by traditional electromagnetic power-generation equipment [9,10,11,12]. The piezoelectric effect lays the physical foundation for mechanical-to-chemical and mechanical-to-energy conversion, which exclusively emerges in non-centrosymmetric crystalline materials. Piezocatalysis employs strain-induced piezopotential to drive interfacial redox reactions, yet pure piezocatalysis generally suffers from relatively low charge-carrier density [13,14].

To circumvent the respective deficiencies of standalone piezocatalysis and standalone photocatalysis, piezo-photocatalysis, which organically combines photoexcitation and piezoelectric polarization, has been put forward as a synergistic composite strategy [15,16,17,18,19]. The built-in piezoelectric electric field serves as an internal charge separator to facilitate the directional migration of photogenerated carriers, suppress rapid recombination, and prolong carrier lifetime. Under simultaneous light and mechanical-force excitation, piezopotential tilts band edges, reconstructs solid–liquid interfacial band bending, optimizes thermodynamic driving forces for redox half-reactions, and modulates the adsorption–desorption behaviors of reaction intermediates. Such coupled systems exhibit promising prospects in wastewater purification, hydrogen-peroxide synthesis, water splitting, and pollutant remediation. Besides catalytic chemical conversion, piezoelectric effects can also be utilized for mechanical-energy collection: piezoelectric nanogenerators transform mechanical deformation into alternating-current electrical signals and supply power for self-powered electronic devices.

Traditional piezoelectric materials mainly fall into two categories: inorganic ferroelectric ceramics and organic piezoelectric polymers [20,21,22,23,24]. Typical inorganic materials possess large piezoelectric coefficients, but suffer from low specific surface area, limited structural tunability and high-temperature synthesis requirements. Benefiting from reticular chemistry, covalent organic frameworks (COFs) are crystalline porous polymers assembled from organic building blocks connected via robust, reversible covalent bonds such as imine, boronate ester, triazine, and β-ketoenamine linkages. Compared with metal–organic frameworks (MOFs), COFs display multiple unique intrinsic advantages for piezoelectric-related applications [25,26,27,28,29,30].

The theoretical prediction of piezoelectric properties for monolayer COFs materials was first reported in 2017 [31]. Since then, accumulating experimental evidence has verified prominent piezoelectric responses in a variety of non-centrosymmetric COF systems [32]. Nevertheless, current research on piezoelectric COFs is still in the early exploratory stage. Although several excellent reviews on general piezocatalysis, piezo-photocatalysis and 2D piezoelectric materials have been published recently [25,29,33,34], they either focus on rigid inorganic piezoelectric systems with limited discussion of COFs-specific electromechanical behaviors or only cover basic material synthesis and characterization of 2D piezoelectric COFs without systematic collation of catalytic mechanisms, multi-scale modification strategies, or full application spectra. To date, a comprehensive review that is entirely dedicated to COFs-based piezocatalysis, piezo-photocatalysis and piezoelectric energy harvesting remains absent.

To explicitly demonstrate the novelty and specific contributions of this review, we benchmark it against the two most closely related prior works: the 2D piezoelectric COFs review by Gu et al. [25] and the polarization engineering in COFs catalysis review by Wang et al. [35]. The key distinctions are summarized across six dimensions, as follows.

Material classes: The 2D piezoelectric COFs review focuses exclusively on 2D layered structures. The polarization engineering review treats piezoelectric effects only as a minor modulation strategy for general catalysis. This work comprehensively covers four categories of COFs-based piezoelectric systems: intrinsic piezoelectric COFs (both 2D and 3D), COFs–polymer composites, single-piezophase heterostructures, and dual-piezophase heterostructures.

Mechanistic depth: Neither prior review systematically dissects COFs-specific flexible-pore electromechanical behaviors or draws a critical distinction between intrinsic lattice dipole polarization and extrinsic electric-field contributions from heterojunction interfaces or polymer matrices. This work elaborates three competing interpretive frameworks for piezocatalysis and analyzes the unique electromechanical properties of flexible porous COFs skeletons.

Characterization methodology: The prior reviews do not address methodological pitfalls specific to piezocatalysis research. This work systematically analyzes piezoresponse force microscopy (PFM) measurement artifacts in soft porous COFs systems and proposes a standardized set of control experiments to rigorously distinguish true piezocatalysis from ultrasound-derived side effects.

Application scope: Existing reviews tend to center on energy harvesting or conventional catalysis, merely providing cursory descriptions of piezoelectric applications. In contrast, this review systematically addresses three primary research areas: piezocatalysis, piezo-photocatalysis, and piezoelectric energy harvesting. It includes six concrete application cases as well as newly developed interdisciplinary directions, such as uranium extraction from seawater and selective modulation of pathogenic microbiomes.

Comparative performance analysis: The prior reviews lack a unified cross-system performance comparison framework, with performance data scattered across individual studies. This work provides two comprehensive summary tables collating experimental conditions, mechanical stress sources and quantitative performance metrics for all representative material systems, and explicitly notes comparability limitations and calls for standardized testing benchmarks.

Unresolved challenges and outlook: Existing reviews offer narrow viewpoints that largely revolve around material synthesis. By contrast, this work pinpoints five core bottlenecks in a systematic manner: insufficient piezoelectric coefficients, unclear reaction mechanisms, barriers in material synthesis and processability, poor practical environmental stability, and device-scale engineering deficiencies. Targeted and feasible outlooks for future research are also put forward.

This review aims to provide a comprehensive and dedicated overview of COFs-based piezocatalysis, piezo-photocatalysis and piezoelectric energy-harvesting devices. Four specific objectives are pursued: (1) to elaborate the fundamental mechanisms and their applicability and inherent limitations for flexible porous COFs lattices; (2) to systematically summarize five major performance-promoting modification strategies across different spatial scales; (3) to detail diverse application scenarios of piezoelectric COFs; and (4) to dissect key bottlenecks constraining further development and propose prospective research suggestions.

### Literature Search and Screening Methodology

We conducted a two-tiered structured literature search across three widely used academic databases: Web of Science Core Collection, Scopus, and Google Scholar. For core studies focused on COFs-based piezoelectric systems, our literature search covered the period from 2017 to October 2026: 2017 is the year of the first theoretical prediction of piezoelectric properties for monolayer COFs, and October 2026 represents when we finished the final literature screening. We paired the search term “covalent organic frameworks” with keywords including “piezocatalysis,” “piezo-photocatalysis,” “piezoelectric,” “piezoelectric nanogenerator,” “mechano-catalysis,” and “vibration catalysis.” For supplementary background references including classical mechanistic frameworks, conventional piezoelectric material benchmarks, and standardized characterization methodologies, we also performed searches with standalone keywords including “piezocatalysis,” “piezo-photocatalysis,” “photocatalysis,” and “polarization engineering in porous catalysts,” with no restriction on the starting year.

Studies were included based on the following criteria:

1. Original peer-reviewed research articles (experimental or theoretical).
2. For core COFs-specific studies: focused on COFs-based piezoelectric materials or composite systems, involving at least one of the three core application domains, and reporting quantitative piezoelectric characterization or catalytic performance data.
3. For supplementary studies: focused on general piezocatalytic/photocatalytic mechanisms, classical piezoelectric material benchmarks, or standardized characterization methodologies.

Studies were excluded if they:

1. Focused exclusively on topics irrelevant to piezoelectric catalysis or energy harvesting.
2. Were conference abstracts, unpublished preprints, or doctoral theses without formal peer review.
3. Represented duplicate publications of the same study.

All eligible studies were systematically collated, categorized, and analyzed to construct the unified framework presented in this review.

## 2. Fundamental Mechanisms of COFs-Based Piezocatalysis, Piezo-Photocatalysis and Piezoelectric Energy Harvesting

Piezocatalysis, piezo-photocatalysis and piezoelectric energy harvesting all stem from the piezoelectric effect of non-centrosymmetric crystalline materials. Under external mechanical stimuli such as ultrasonic vibration, fluid shear and cyclic compression bending, lattice deformation induces spatial separation of cation–anion charge centers and generates macroscopic piezopotential. Unlike rigid inorganic piezoelectric crystals, flexible porous COFs skeletons exhibit distinctive mechanochemical responses originating from dynamic linker torsion, bond-angle bending, and interlayer slippage. These COFs-specific structural features modify polarization magnitude, charge-screening behavior and strain-transfer efficiency, and cannot be fully described by classical piezoelectric theories developed for dense inorganic solids. This section elaborates on three competing interpretive frameworks for piezocatalysis, conventional photocatalytic mechanisms, and the piezo-photocatalytic synergistic effect, together with the unique electromechanical behaviors of flexible-pore COFs skeletons, as well as the operating principle of COFs-constructed piezoelectric nanogenerators for mechanical-energy collection. Special attention is paid to their applicable scenarios and inherent limitations in porous organic-framework systems.

### 2.1. Core Catalytic Mechanisms

#### 2.1.1. Intrinsic Piezocatalytic Mechanism

The intrinsic piezocatalytic mechanism refers to redox reactions driven exclusively by strain-generated piezopotential in the absence of light irradiation. For COFs, macroscopic net polarization depends strongly on long-range crystallinity, dipole-domain orientation, defect density, and interlayer stacking geometry. Even with strong individual molecular dipoles, poor crystallinity or random dipole orientation will lead to mutual dipole cancellation and negligible net piezoresponse. When non-centrosymmetric COFs lattices undergo mechanical deformation, the spatial separation of cation and anion charge centers produces built-in dipole moments and surface piezopotential. The resulting potential difference provides the thermodynamic driving force for interfacial charge transfer. Electrons and holes originating from lattice polarization migrate to COFs surfaces, triggering reduction and oxidation reactions, respectively. The fundamental principles of piezocatalysis include the energy-band theory, screening-charge theory and displacement-current theory.

**Figure 1:**
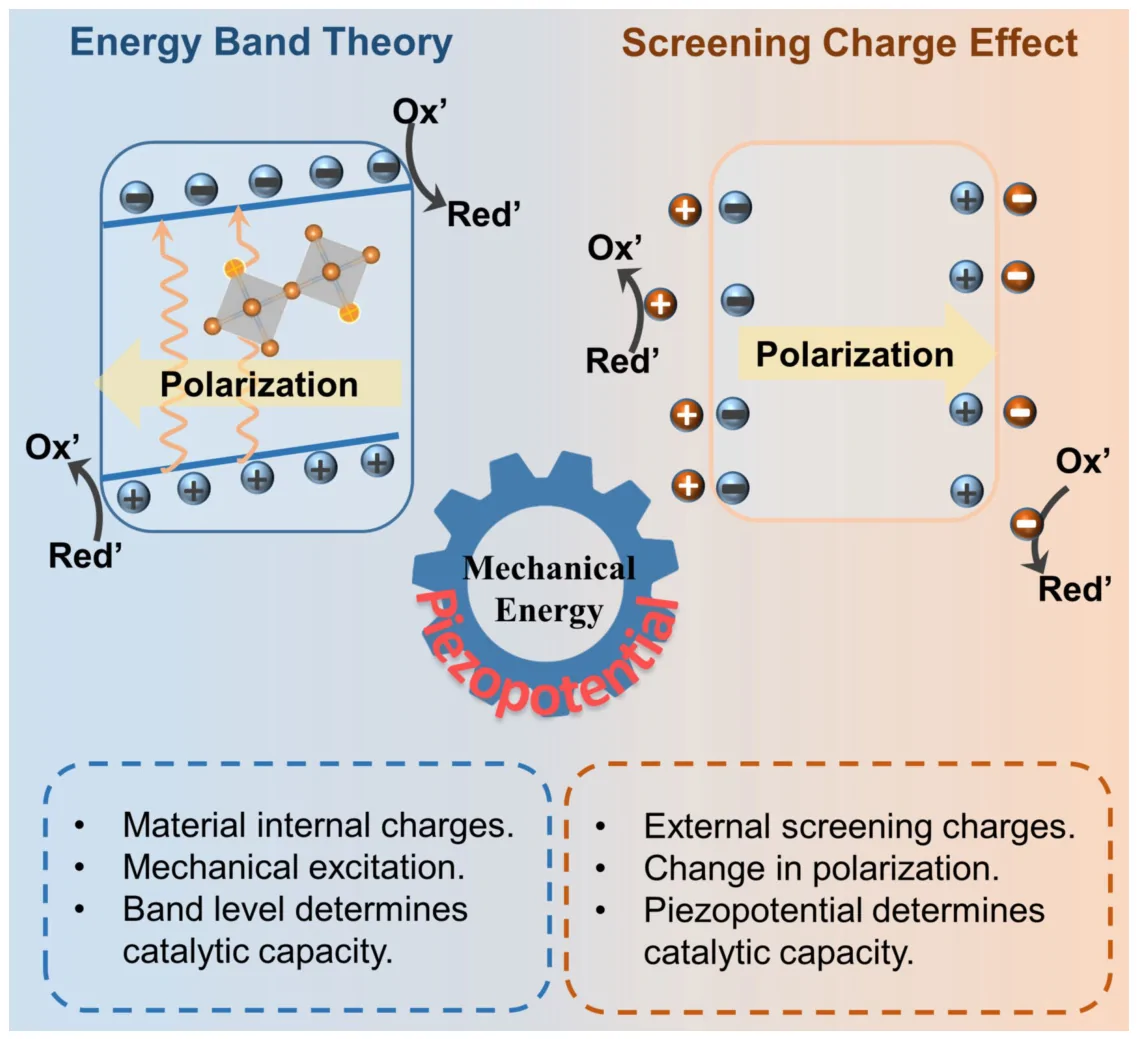
Schematic comparison of the energy-band theory and screening-charge-effect theory for piezocatalysis. Reprinted with permission from Ref. [36]. Copyright 2021, John Wiley & Sons.

The energy-band theory of piezocatalysis has been invoked to interpret the framework of photocatalysis and incorporates piezoelectric principles to interpret redox behaviors in piezocatalysis [36] (Figure 1). In conventional photocatalytic water splitting, incident light with energy greater than or equal to the semiconductor bandgap excites electrons from the valence band to the conduction band, which further drives water reduction for hydrogen production. By analogy, piezocatalytic energy-band theory suggests that the capability of a piezocatalyst to initiate a target chemical reaction is largely governed by the band-edge positions of its conduction and valence bands. Upon mechanical stress, piezoelectric materials become polarized and generate piezopotential. Within this theoretical framework, piezopotential substantially modulates band-edge positions, tilting both conduction and valence bands simultaneously. Band bending facilitates the migration and accumulation of separated charge carriers on material surfaces, thereby enabling catalytic redox reactions.

The screening-charge theory has been invoked to interpret how mechanical loading periodically disrupts the equilibrium between piezoelectric polarization and surface screening charges, which generates free charge carriers for subsequent redox reactions (Figure 1) [36]. In highly porous COFs, enlarged pore-surface area amplifies surface charge-screening effects, partially neutralizing bulk polarization and lowering the effective piezopotential available for catalysis. Under electrostatic equilibrium, bound polarization charges inside piezoelectric materials are neutralized by external screening charges, yielding zero net internal polarization intensity. For piezocatalytic water splitting, applied tensile or compressive stress alters the internal polarization intensity (P) and breaks the surface charge equilibrium, which gives rise to piezopotential. Positively charged sites on the catalyst surface react with water-phase oxygen species to produce O_2_, while negative charges combine with H^+^ to yield H_2_. Water molecules act as the primary reactant. At positively charged surface sites, water undergoes oxidation to produce oxygen: $2H_{2}O\;\to \;O_{2}\;+\;4H^{+}+\;4e^{-}$. At negatively charged sites, protons are reduced to generate hydrogen:$\;2H^{+}+\;2e^{-}\to H_{2}$. Distinct from energy-band theory, the screening-charge mechanism highlights the critical roles of piezopotential and surface screening phenomena.

Originating from Maxwell’s equations, displacement-current theory complements the previous two mechanistic models and provides a unified interpretation of piezocatalytic charge production for both semiconductor-type and insulating piezoelectric systems [37,38,39]. Cyclic mechanical strain reconfigures lattice dipole moments and builds time-changing polarization fields, producing equivalent displacement current that powers interfacial electron migration. Directional free conduction current fails to emerge within the material bulk. Time-varying displacement current instead transports charge carriers toward surface active sites to initiate catalytic redox processes.

Distinct from photocatalysis, charge carriers in pure piezocatalysis are produced via mechanical lattice polarization rather than photon absorption. Carrier density is highly dependent on external-strain intensity, intrinsic skeleton dipole density, and charge-separation efficiency. Under mild mechanical excitation, most COFs piezocatalysts deliver relatively low carrier concentrations, leading to moderate reaction rates. Porous COFs contain abundant surface trap states. These trap sites can capture piezogenerated carriers and suppress partial charge recombination, yet they may also consume active carriers before redox reactions take place.

Notably, all the above intrinsic piezocatalytic mechanisms rely on lattice polarization-driven charge generation under mechanical strain. However, in most experimental studies, ultrasonic vibration is employed as the mechanical excitation source, which may introduce additional non-piezoelectric side effects that contribute to catalytic activity, leading to potential overestimation of true piezocatalytic performance. These ultrasound-related effects include sonochemical heating, acoustic cavitation-induced water pyrolysis, and sonoluminescence, which are fundamentally distinct from true piezocatalysis.

True piezocatalysis originates from non-centrosymmetric lattice deformation that generates intrinsic polarization charges and drives interfacial redox reactions. It is a solid-state electromechanical coupling effect that depends on the crystal symmetry and piezoelectric properties of the catalyst itself. In contrast, ultrasound-related side effects arise from the interaction between acoustic waves and the liquid medium: acoustic cavitation generates localized high-temperature and high-pressure microenvironments that cause water pyrolysis to produce radicals, while sonoluminescence may emit weak light that excites photocatalytically active materials. These effects do not require the catalyst to have piezoelectric activity, and they can occur even with centrosymmetric or amorphous materials under ultrasonic irradiation.

To rigorously distinguish true piezocatalysis from ultrasound-related effects, a set of controlled experiments must be implemented:

1. Temperature control experiments: monitoring reaction temperature changes and conducting isothermal control tests to exclude thermal contribution from sonochemical heating.
2. Non-piezoelectric material control: testing centrosymmetric isostructural materials or amorphous counterparts under identical ultrasonic conditions to quantify the contribution from pure sonochemical effects.
3. Radical quenching and product identification under different excitation modes: comparing product species and radical types between piezocatalytic and pure sonochemical systems.
4. Frequency/power-dependent activity tests: verifying that the activity variation correlates with piezoelectric resonance rather than simply increasing monotonically with ultrasonic power.

It should be emphasized that for COFs-based systems, their high porosity and large specific surface area may enhance mass transfer and amplify the contact between reactants and cavitation sites, which may further exaggerate the contribution of ultrasound side effects. Therefore, strict control experiments are indispensable for reliable evaluation of COFs piezocatalytic performance.

Notably, piezoresponse force microscopy (PFM) is the most widely used technique for nanoscale piezoelectric characterization, but its results must be interpreted with caution, especially for soft, porous COFs systems. The measured PFM amplitude and phase signals do not exclusively originate from intrinsic piezoelectric lattice deformation. Non-piezoelectric contributions including electrostatic tip-sample interaction, universal electrostriction, ion migration-induced electrochemical swelling, and viscoelastic indentation of the soft organic framework can all contribute to the detected displacement signal. For COFs materials in particular, their low elastic modulus makes them prone to tip-induced indentation artifacts, while their high surface charge density and abundant surface functional groups may amplify electrostatic and electrochemical contributions. These effects can lead to overestimation of the intrinsic piezoelectric coefficient, and in extreme cases may produce false-positive piezoelectric signals even for centrosymmetric samples. Therefore, PFM hysteresis loops alone are not sufficient to definitively prove piezoelectricity: complementary control experiments and cross-validation with other techniques are required.

Fundamental mechanistic debates among energy-band theory, screening-charge theory and displacement-current theory are still not fully resolved for porous organic COFs piezocatalytic systems, as summarized in a recent perspective article [39]. It should be emphasized that these three frameworks represent competing interpretive hypotheses to rationalize piezocatalytic phenomena. Although macroscopic experimental observations, including mechanical-stimulated charge generation and redox reactions, are well established, definitive operando experimental proof to judge which model predominantly governs COFs-based piezocatalysis remains scarce. Ultrasound-derived side effects, variable surface adsorption behavior and defect-related trap states further complicate the assignment of measured catalytic activity purely to piezoelectric polarization.

#### 2.1.2. Conventional Photocatalytic Mechanism

**Figure 2:**
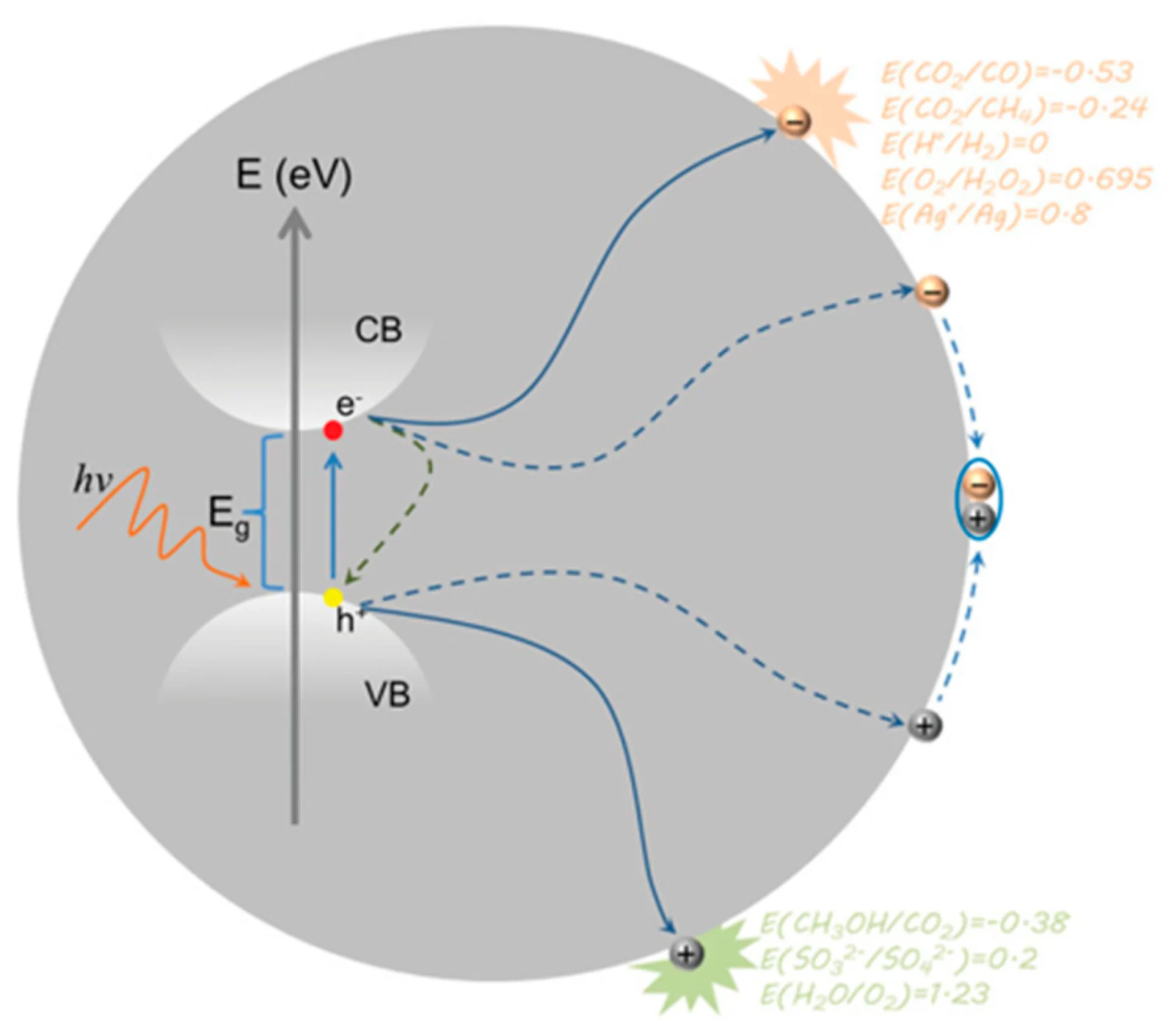
Schematic diagram of photocatalyst excitation mechanism. Reprinted with permission from Ref. [44]. Copyright 2022, John Wiley & Sons.

Conventional photocatalysis relies entirely on photon excitation to generate electron–hole pairs [40,41,42,43]. After absorbing photons with energy exceeding the bandgap value, electrons transit from the valence band to the conduction band, leaving holes in the valence band. The separated electrons and holes migrate toward the material surfaces and participate in reduction and oxidation reactions. Regrettably, over 90% of photogenerated carriers recombine within picosecond-to-nanosecond timescales in most semiconductor photocatalysts, dissipating photon energy as heat or fluorescence instead of driving target surface conversions. Without mechanical-field assistance, photogenerated-carrier separation mainly depends on built-in potentials derived from heterojunctions or surface defects, and the whole system cannot function under dark conditions. This mechanism acts as an important control baseline for evaluating piezo-photocatalytic synergistic effects (Figure 2) [44].

#### 2.1.3. Piezo-Photocatalytic Synergistic Mechanism

**Figure 3:**
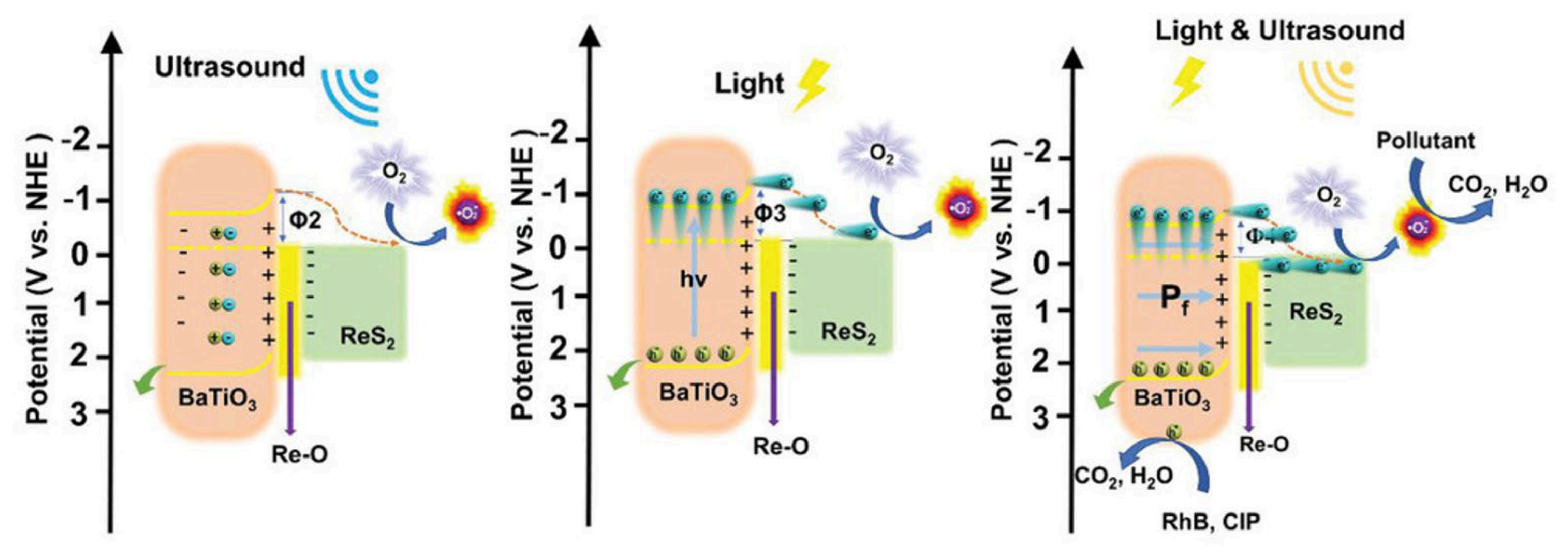
Schematic illustration of the synergy of photocatalysis and piezotronic effects on a piezo-photocatalysis system. Reprinted with permission from Ref. [46]. Copyright 2022, John Wiley & Sons.

Piezo-photocatalysis integrates photoexcitation and strain-induced piezoelectric polarization [16,45]. Figure 3 schematically compares the band-alignment and charge-separation behaviors of photocatalysis, piezocatalysis, and piezo-photocatalysis [46]. Strain-produced piezopotential serves as an extra internal electric field, tilting band edges and reconstructing solid–liquid interfacial band bending. Such band-structure modulation promotes the directional migration of photogenerated electrons and holes, effectively inhibiting ultrafast carrier recombination and extending carrier lifetime. Under co-excitation of light and mechanical force, adjusted band alignment optimizes thermodynamic driving forces for redox half-reactions and regulates the adsorption–desorption properties of key reaction intermediates. Compared with standalone piezocatalysis or standalone photocatalysis, this synergistic system achieves prominently improved conversion efficiency for water splitting, pollutant degradation, and CO_2_ photoreduction.

Beyond individual mechanistic frameworks, the overall piezoelectric and catalytic performance of COFs is collectively governed by a chain of interrelated structural parameters spanning from molecular to macroscopic scales, and their interactions are far from simply additive.

At the molecular level, broken inversion symmetry generates intrinsic molecular dipoles, but dipole magnitude alone does not directly translate to macroscopic piezoelectric output. Dipole orientation ordering, which is largely determined by long-range crystallinity, plays a more decisive role: moderately asymmetric monomers that maintain high crystallinity often deliver stronger macroscopic polarization than highly asymmetric monomers that severely disrupt lattice ordering and lead to random dipole misalignment. This results in a non-monotonic relationship between the degree of symmetry breaking and overall piezoresponse, rather than a linear positive correlation.

At the lattice level, permanent porosity is a defining advantage of COFs for catalytic applications, as it provides large specific surface area, abundant exposed active sites and accelerated mass transport. However, high porosity also introduces inherent trade-offs for piezoelectric performance: abundant pore surfaces strengthen surface charge-screening effects, which partially neutralize polarization charges and reduce the effective piezopotential driving force for redox reactions. In addition, porous frameworks generally exhibit lower mechanical stiffness than dense inorganic crystals, which may dissipate external stress and reduce strain-transfer efficiency to the inner skeleton.

At the electronic level, charge-transport efficiency determines how many polarization-generated carriers can reach the surface to participate in catalytic reactions before recombination. While high crystallinity facilitates long-range charge migration, abundant surface trap states associated with high porosity may capture carriers and suppress charge delivery. Moreover, mechanical deformation of the flexible skeleton not only generates polarization, but also modulates band structure and charge hopping dynamics: moderate torsion enhances charge separation, while excessive deformation may introduce deep trap states and accelerate carrier recombination.

Taken together, optimizing piezoelectric COFs performance requires balanced modulation of multiple structural parameters rather than maximization of any single parameter. The synergy among symmetry breaking, dipole ordering, appropriate porosity, efficient charge transport and suitable mechanical deformability is the key to achieving both high piezoelectric polarization and high catalytic activity.

### 2.2. Operating Principle of COFs-Based Piezoelectric Nanogenerators for Mechanical-Energy Harvesting

Different from piezocatalysis occurring at solid–liquid interfaces, COFs-based piezoelectric nanogenerators (PENGs) realize mechanical-to-electric energy harvesting within solid-state composite films and deliver alternating-current outputs upon mechanical deformation [29]. Typically, non-centrosymmetric COFs fillers are blended into insulating polymer matrices to construct flexible functional membranes. Applied strain deforms the COF lattice and generates bound polarization charges. Variations in strain propel free-electron flow through the external circuit and produce measurable voltage and current. Once mechanical stress is released, the lattice reverts to its initial state and electrons flow in the reverse direction.

**Figure 4:**
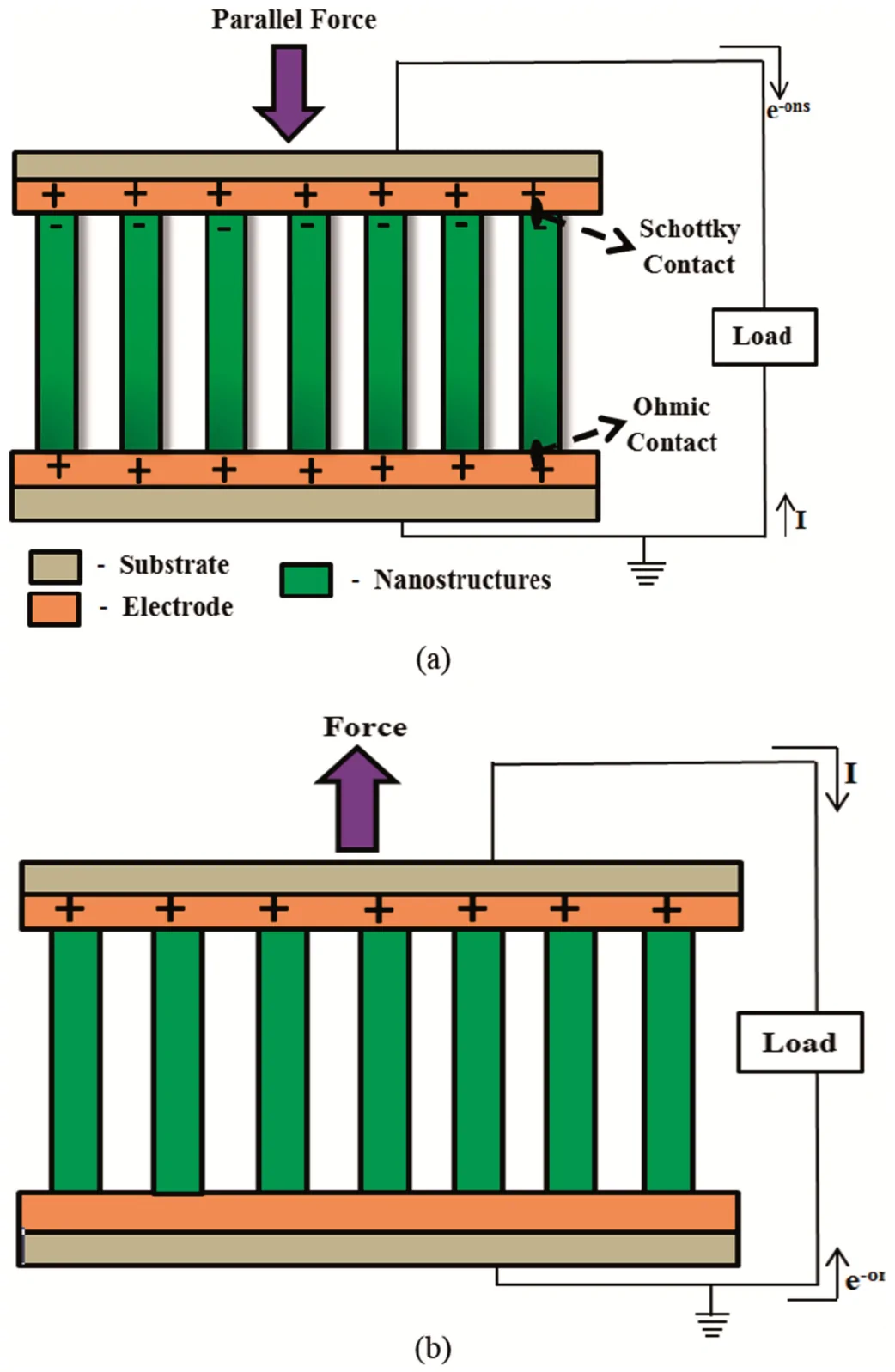
Schematic of PENG. (**a**) After the application of a force parallel to the NG axis. (**b**) After the removal of the force. Reprinted with permission from Ref. [47]. Copyright 2017, Royal Society of Chemistry.

The core physical principles of piezoelectric nanogenerators can be exemplified by the classic vertically aligned nanostructured model system [47,48,49,50,51]. Figure 4a corresponds to the compressive loading process, while Figure 4b represents the unloading process. For conventional PENGs built from vertically aligned nanostructures, compressive loading along the nanostructure axis triggers uniaxial mechanical deformation that generates intrinsic electric dipole moments through the direct piezoelectric effect. This gives rise to negative electrostatic potential at the nanostructure tip and raises the local Fermi-level energy. Driven by the resulting potential gradient, electrons drift from the upper tip toward the distal bottom end of the nanostructure. Arising from the coupling between semiconductor behavior and piezoelectric response, the piezotronic effect facilitates the formation of a Schottky contact. This barrier inhibits parasitic internal electron leakage inside the nanostructure and operates as an effective charge pump, driving electron transport across the external circuit until a state of charge equilibrium is reached. Upon removal of the applied mechanical load, piezoelectric-driven polarization dissipates abruptly. Charge carriers that have accumulated at the bottom electrode travel backward toward the top electrode via the external circuit to cancel out the potential offset, yielding an opposite-polarity voltage peak and consequently producing alternating-current outputs.

Despite sharing the fundamental piezoelectric origin with the abovementioned model system, COFs–polymer composite systems possess several unique features inherited from the intrinsic porous nature of COFs materials. The inherent pore architecture of COFs introduces internal void domains within composite films, which further modulates the dielectric properties and mechanical robustness of the final device. In particular, the dispersion state and mass loading of COFs fillers exert a strong influence on the overall piezoelectric output performance. For COFs-based PENG devices, key performance-determining factors include the strain-transfer efficiency from the polymer matrix to embedded COFs crystallites, electrode interfacial contact quality, and the mechanical flexibility of the whole membrane. To date, most reported COFs-based PENG prototypes have achieved considerable open-circuit voltage, yet suffer from relatively low output-current amplitude. This represents the major bottleneck restricting their practical implementation for self-powered sensors and wearable electronic applications.

Notably, essential differences exist between piezocatalysis and COFs-based PENG operation. Piezocatalysis proceeds in liquid-phase solid–liquid interfacial environments, while nearly all COFs-based PENGs operate in all-solid-state film configurations. Given the large divergence in stress-transfer conditions for these two scenarios, experimental observations and conclusions derived from liquid-phase piezocatalytic measurements cannot be directly extrapolated to solid-state energy-harvesting devices.

## 3. Performance-Boosting Strategies for Piezoelectric COFs

As-synthesized COFs often have high-symmetry centrosymmetric structures with negligible piezoelectric response. The core design principle is breaking crystallographic inversion symmetry to construct molecular/local dipoles and amplify piezopolarization. Five mainstream strategies are summarized: building-block molecular design, defect engineering, morphology modulation, single-atom anchoring, and heterostructure construction. Each strategy introduces symmetry breaking at different spatial scales: molecular scale, local lattice scale, micro/nanoscale, and interface scale. Various structural modification strategies have been developed to boost the piezoresponse of COFs-based materials. These strategies act at different spatial scales ranging from molecular skeleton to heterointerface, as summarized in Table 1. A recent review built an explicit hierarchy of polarization descriptors for low-dimensional piezoelectric materials and emphasized the critical requirement to strictly separate intrinsic lattice polarization from conventional heterojunction built-in electric-field effects [35]. While that review provides generalized classification for 2D piezoelectric systems, the present review delivers several distinct new insights specifically for porous flexible COFs platforms. First, we highlight COFs-unique pore-dominated phenomena including surface charge screening, dynamic linker torsion and interlayer slippage, which strongly modify macroscopic piezoresponse, but are rarely discussed for dense inorganic piezoelectric crystals. Second, we systematically sort five multi-scale modification strategies spanning from molecular scale, local lattice scale, and micro-nanoscale to heterointerface scale, and explicitly elaborate unavoidable performance-structure trade-offs for each route. Throughout Section 3 and Section 6, we repeatedly distinguish intrinsic COFs lattice dipole polarization from extrinsic contributions originating from heterojunction interfaces or polymer matrices, avoiding misattribution of enhanced catalytic activity solely to COFs piezoelectricity.

### 3.1. Building-Block Molecular Design

Molecular-level structural modification is the most fundamental approach to introducing non-centrosymmetric space groups and intrinsic lattice dipoles by selecting twisted, helical, or polar-group-functionalized monomers. By rationally tuning monomer geometry and electronic configuration, intrinsic molecular dipoles can be embedded into COF skeletons during reticular synthesis, avoiding post-treatments such as electrical poling required for many polymer piezomaterials. Optimal piezoresponse usually emerges at a moderate symmetry-breaking degree that balances molecular-dipole strength and lattice long-range order, instead of pursuing maximized molecular-dipole magnitude alone. For COFs constructed from twisted or polar building blocks, PFM characterization can exhibit enhanced electromechanical-response signals relative to their respective structural reference analogues. It is worth noting that these PFM data only reflect relative signal-magnitude trends and cannot independently confirm intrinsic lattice piezoelectricity for soft porous COFs, regardless of whether the reference analogues are centrosymmetric or non-centrosymmetric. Cross-validation with other characterization tools is therefore required.

**Figure 5:**
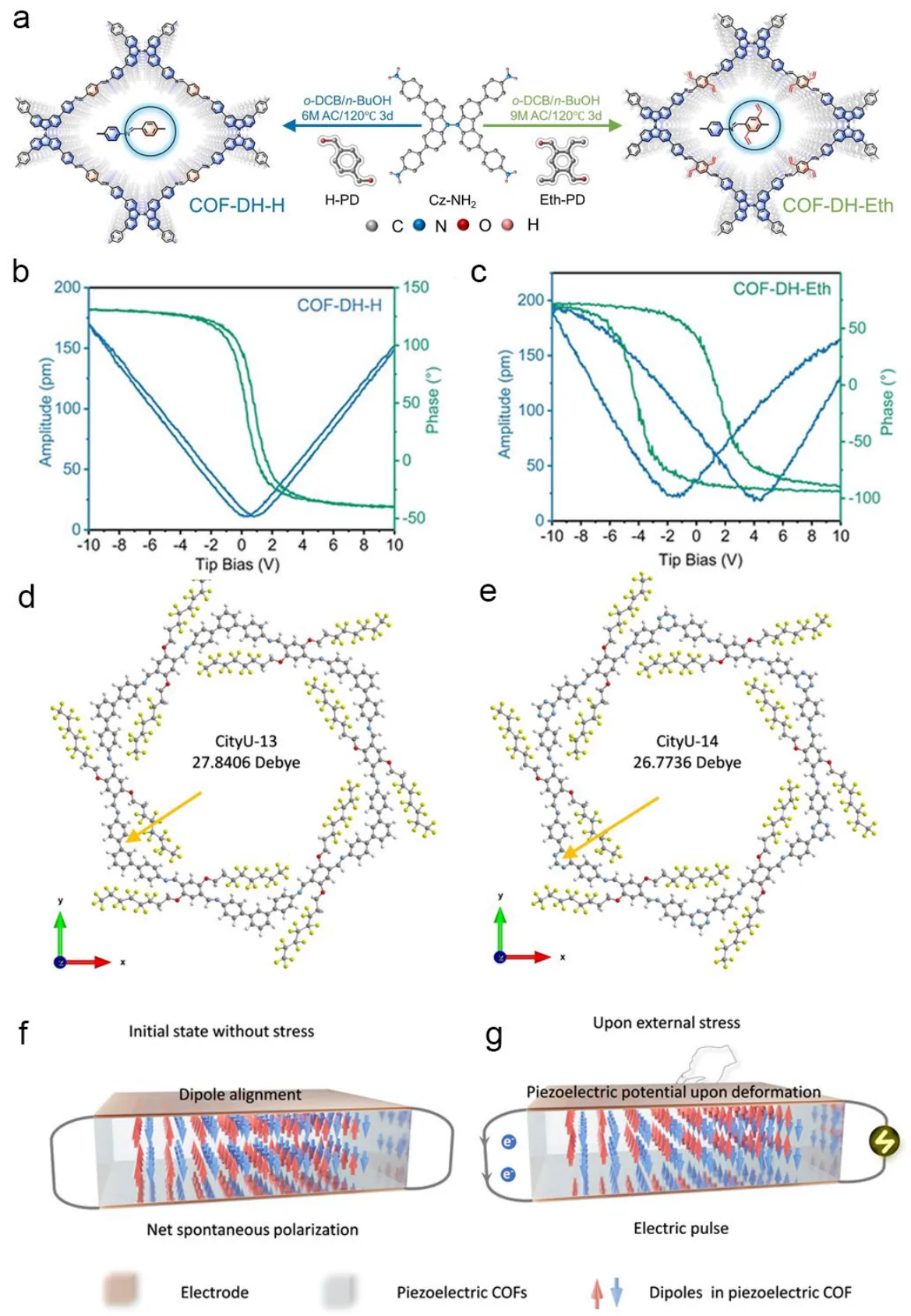
(**a**) Synthetic routes of COF-DH-H, and COF-DH-Eth. The phase hysteresis loop and amplitude butterfly loop of (**b**) COF-DH-H and (**c**) COF-DH-Eth (reprinted with permission from Ref. [52]. Copyright 2025, John Wiley & Sons). (**d**,**e**) Density functional theory (DFT) simulation of dipole moments for CityU-13 and CityU-14. The arrow shows the conventional direction of dipole moment vector. (**f**) Schematics of dipole alignment and net spontaneous polarization in COFs without stress. (**g**) Schematic illustration of piezoelectricity with electric pulse due to the separation of positive/negative charges upon stress (reprinted with permission from Ref. [53]. Copyright 2024, John Wiley & Sons).

Twisted building units: Bicarbazole and bifluorenylidene twisted monomers are representative building blocks with non-planar conformations. Liu and coworkers scientifically designed bicarbazole-based Cz-COFs [52]. The non-planar twisted conformation of bicarbazole motifs breaks planar centrosymmetry and generates intrinsic molecular dipoles within the covalent framework, which builds efficient piezopolarization electric fields. On this basis, they originally proposed a piezocatalysis–photocatalysis coupling strategy toward hydrogen peroxide production. Benefiting from multiple charge-transfer pathways and the synergistic effect of the piezopolarization electric field driven by twisted-structure-originated dipoles, Cz-COFs exhibit greatly promoted separation of photogenerated charge carriers. Among them, ethylene-modified COF-DH-Eth delivers a record-high hydrogen peroxide-production rate. Twisted configurations destroy planar centrosymmetry and produce inherent molecular dipoles along specific crystallographic directions. The dihedral angle between two aromatic segments in twisted monomers can be adjusted by steric hindrance of side groups, further regulating dipole magnitude and skeleton flexibility, so as to modulate the overall piezoelectric response. For instance, COF-DH-Eth delivers a piezoelectric coefficient value surpassing commercial PVDF. As displayed in Figure 5a, the synthetic route from COF-DH-H toward ethylene-functionalized COF-DH-Eth demonstrates side-group steric-hindrance modulation on twisted bicarbazole skeletons. PFM amplitude-phase hysteresis loops in Figure 5b,c provide comparative experimental signals suggesting enhanced electromechanical response after ethylene modification. It should be noted that such PFM hysteresis signals alone cannot serve as definitive proof of intrinsic lattice piezoelectricity for soft porous COFs materials, as discussed in Section 2. Additional cross-validation by complementary techniques such as second-harmonic generation or transient piezoelectric current measurements is required to confirm intrinsic piezoelectric origins. These PFM results are used here for relative trend comparison within this material series.

Polar-group/fluorinated-alkyl-chain modification: Gu and co-workers reported that CityU-13 and CityU-14 feature fluorinated alkyl side chains grafted onto the COF backbones [53]. Highly electronegative C–F bonds generate strong dipole moments along the side-chain direction, and such oriented local dipoles collectively contribute to the macroscopic piezoelectricity of the whole framework. Corresponding theoretical dipole calculations for CityU-13 and CityU-14 are presented in Figure 5d,e. Large molecular dipole moments originate from oriented fluorinated side chains, which endow the COF framework with collective macroscopic polarization. The conceptual diagrams in Figure 5f,g further generalize the underlying physical picture: ordered molecular dipoles give rise to net spontaneous polarization under initial unstressed conditions, and external mechanical deformation triggers piezoelectric potential and electric-pulse output. Assembled piezoelectric nanogenerators achieve high open-circuit voltage. Apart from fluorinated groups, cyano, hydroxyl, and sulfonic polar groups can also introduce local dipoles to boost piezoresponse, while excessive polar side chains may undermine crystallinity and porosity, requiring trade-off optimization.

Propeller-like/helical non-planar building blocks: Ghosh et al. reported a metal-free TAPA-PDA COF, in which conformationally flexible TAPA donor units couple with PDA acceptor moieties [54]. The intrinsic twisted propeller motif disrupts crystal symmetry by steric repulsion among multi-arm aromatic segments, which induces permanent molecular dipoles and yields a prominent piezoresponse. Exfoliated nanosheets achieve considerable piezocatalytic water-splitting hydrogen production. Propeller-type units possess multiple aromatic arms diverging from a central core; steric repulsion prevents a planar conformation and guarantees permanent molecular chirality or a dipole. Helical building blocks can further introduce a chiral non-centrosymmetric lattice for the construction of piezoelectric COFs, where helical conformation geometry dominates the orientation and magnitude of lattice dipoles.

Donor–acceptor (D–A) skeletons: Benzothiadiazole acts as a strong acceptor unit to construct TP-BT-0F-COF [55]. D–A intermolecular interactions create strong in-plane dipoles and enhance piezoresponse for efficient piezo-photocatalytic water splitting. Liang and co-workers further proposed constructing robust in-plane dipoles by integrating symmetry-broken benzothiadiazole acceptors with β-ketoenamine linkages. A series of TP-BT-xF-COFs were synthesized, where symmetry-broken D–A interactions lock strong intrinsic dipole moments into COF skeletons to reinforce piezoresponse. Under simultaneous light–ultrasound excitation, TP-BT-0F-COF can realize the concurrent generation of hydrogen and hydrogen peroxide with considerable yields. These works underscore that engineering ferroelectric dipole ordering and strong intraskeleton D–A interactions is a viable strategy to advance the intrinsic piezoelectric performance of COFs at the molecular level.

Zwitterionic ionic-displacement-type building blocks: Yan and co-workers developed zwitterionic COFs as a distinctive subclass for piezocatalysis, wherein atomic-level ionic-displacement polarization is incorporated into the COF backbones [56]. Taking SQ-TAPT (zwitterionic squaric-acid-based covalent organic framework derived from 4,4′,4″-(1,3,5-triazine-2,4,6-triyl)-trianiline (TAPT)) as a typical example, positively and negatively charged moieties are covalently integrated within the framework. The large Born effective charges originating from ionic bonding enable substantial relative atomic displacement under external mechanical strain, generating greatly amplified dipole moments and macroscopic piezoelectric response. SQ-TAPT exhibits much stronger piezoelectric performance compared with its isostructural neutral counterpart terephthaldehyde and 4,4′,4″-(1,3,5-triazine-2,4,6-triyl)trianiline-based covalent organic framework (PDA-TAPT). Besides enhancing polarization, zwitterionic skeletons endow COFs with superior hydrophilicity, optimizing water-molecule adsorption and accelerating the Volmer step of hydrogen evolution. Theoretical calculations reveal that the zwitterionic skeleton optimizes hydrogen-adsorption behavior and improves intrinsic hydrogen-evolution catalytic kinetics. Without any co-catalyst, SQ-TAPT achieves prominent piezocatalytic hydrogen evolution performance, far outperforming neutral PDA-TAPT under identical ultrasonic excitation conditions. Nevertheless, strong Coulomb repulsion among oppositely charged fragments in zwitterionic COFs usually deteriorates long-range crystallinity and reduces Brunauer–Emmett–Teller (BET) specific surface area, which constitutes a major trade-off for this molecular-design strategy and should be carefully balanced in future monomer design.

In summary, building-block molecular design achieves intrinsic symmetry breaking at molecular scale without post-treatment poling. This strategy directly tunes molecular dipole magnitude and skeleton flexibility to enhance piezoresponse. The major inherent trade-off lies in the competition between strong molecular dipoles and long-range crystallinity: highly twisted or polar monomers can generate large unit-cell dipoles, yet may disrupt ordered packing and weaken net macroscopic polarization. The generalized design principle is to pursue a moderate symmetry-breaking degree that balances dipole strength and crystallinity, rather than simply maximizing molecular dipole magnitude. Zwitterionic building blocks represent a promising sub-route to realize large ionic-displacement polarization, where mitigating Coulomb-repulsion-induced crystallinity loss remains an important design challenge.

From a comparative perspective, twisted-unit COFs generally deliver good piezocatalytic H_2_O_2_ performance, but often suffer from moderate piezoelectric coefficients. Polar-group-grafted COFs are well suited for piezoelectric nanogenerator devices, yet heavy side-chain functionalization may reduce pore volume and accessible surface area. Zwitterionic COFs produce large ionic-displacement-driven polarization and show advantages for piezocatalytic hydrogen evolution. Nevertheless, Coulomb repulsion frequently degrades crystallinity compared with twisted or polar-group-modified analogues. Each subclass faces its characteristic bottleneck, and no single building-block motif can simultaneously maximize piezoelectric coefficient, crystallinity, and porosity.

### 3.2. Defect Engineering

Missing-linker vacancies are artificially introduced via reversible imine-bond kinetics, triggering local lattice distortion, breaking local inversion symmetry, and generating localized polarization domains. Different from global symmetry breaking achieved by monomer design, defect engineering generates symmetry breaking in nanoscale local regions while maintaining the overall crystal framework of the COFs. Defect sites are simultaneously potential catalytically active centers, enabling simultaneous polarization improvement and activity promotion.

**Figure 6:**
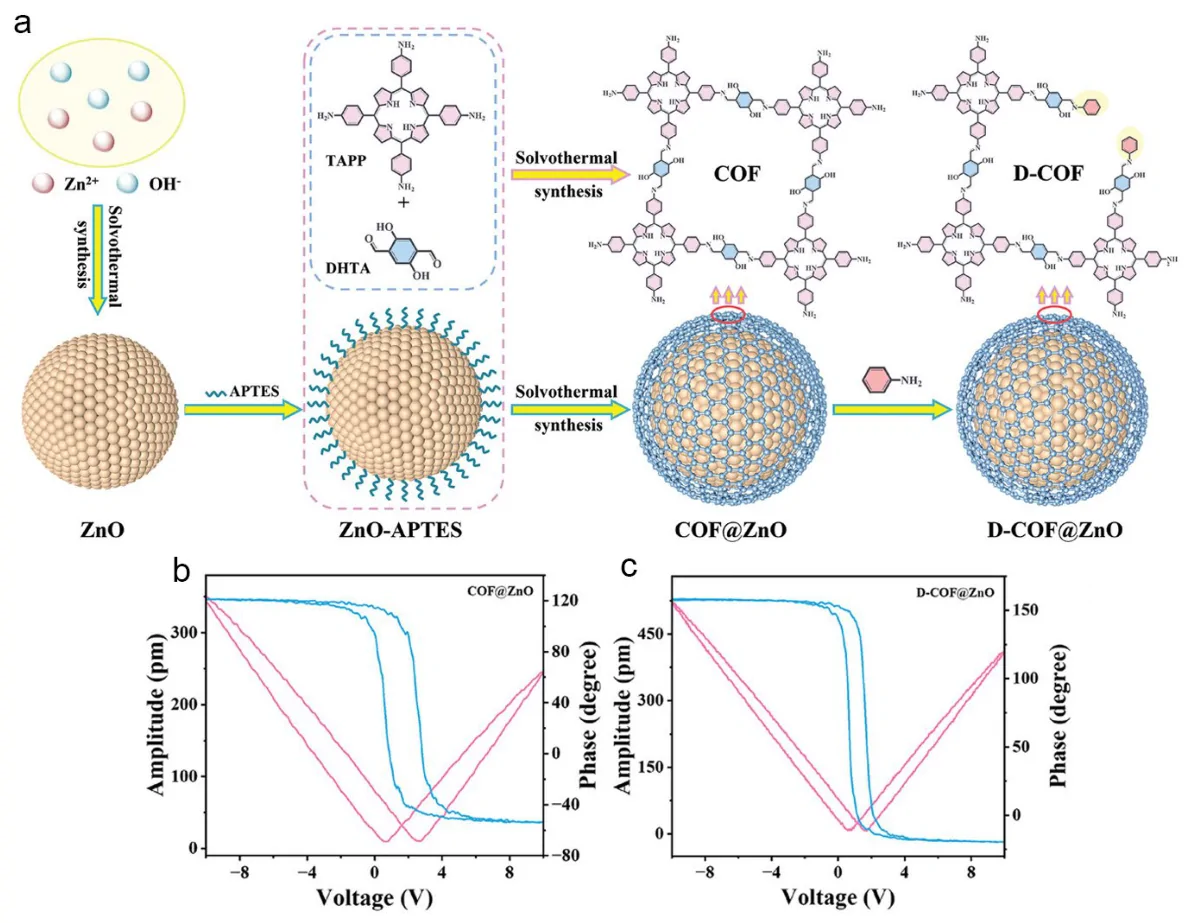
(**a**) Schematic illustration of the synthesis of defect heterogeneous engineering D-COF@ZnO. Piezoresponsive amplitude curve and phase curve of (**b**) COF@ZnO and (**c**) D-COF@ZnO (reprinted with permission from Ref. [57]. Copyright 2024, John Wiley & Sons).

A typical example is the D-COF@ZnO heterojunction [57]. Aniline competitive coordination creates missing-linker defects within COF layers. PFM characterizations demonstrate that defect modification greatly elevates surface piezopotential and transient piezoelectric current. Figure 6a depicts the step-by-step synthetic procedure for pristine COF@ZnO and the defect-engineered D-COF@ZnO heterostructure, where missing-linker vacancies are introduced by aniline competitive coordination. The PFM amplitude-phase hysteresis loops of COF@ZnO and D-COF@ZnO are displayed in Figure 6b and Figure 6c, respectively. Apparently, D-COF@ZnO delivers a larger piezoelectric amplitude signal, confirming that missing-linker defects strengthen local piezoresponse. Defect-containing samples exhibit more saturated PFM hysteresis loops, indicating enhanced local polarization. These PFM results only reflect relative signal trends under identical test conditions. Quantitative piezoelectric coefficients require bulk-scale complementary characterization to rule out electrostatic and viscoelastic artifacts originating from surface defects in porous COFs. Nevertheless, purely relying on building-block design still faces practical challenges: introducing large twisted or propeller-shaped monomers often increases steric hindrance during COF crystallization, which may reduce material crystallinity. While highly asymmetric monomers generate stronger molecular dipoles at the unit cell level, the accompanying loss of long-range order can reduce dipole alignment and thus decrease macroscopic piezoelectric polarization. Balancing non-centrosymmetric dipole construction and high crystallinity remains a key molecular design challenge. For zwitterionic COFs, how to mitigate Coulomb-repulsion-induced crystallinity loss while retaining strong ionic displacement polarization is an open research question.

In summary, defect engineering introduces local symmetry-breaking polarization at lattice scale while largely preserving the overall COF framework. Moderate-density missing-linker vacancies create local distorted coordination environments, generate localized dipoles and simultaneously supply extra catalytic active sites. Its main limitation is that excessive defect concentration will destroy long-range crystallinity, reduce charge mobility and degrade chemical-mechanical stability. The generalized design principle is to precisely regulate defect density: introducing appropriate local lattice distortion for extra polarization, while avoiding over-generation of disorder that impairs framework integrity. Edge defects and grain boundaries in polycrystalline COF powders should also be considered as additional sources of local symmetry breaking.

Compared with global symmetry breaking via building-block modification, defect engineering only generates local polarization domains and retains the original backbone structure of COFs. This avoids severe synthetic difficulties brought by highly twisted monomers, but defect quantity and distribution are hard to quantify and control experimentally. Different from intrinsic dipoles originating from molecular skeletons, defect-induced polarization is always accompanied by abundant surface trap states, which can either promote or suppress charge-carrier transport. Thus, defect-engineered COFs cannot be directly benchmarked against intrinsically non-centrosymmetric COFs without careful quantitative defect characterization.

### 3.3. Morphology Modulation

Adjusting micro-nanostructure and dimensionality modifies mechanical deformability and stress-response behaviors without altering chemical composition and molecular skeleton structure. The same COFs chemical composition can exhibit drastically different piezoresponse when fabricated into nanotubes, nanosheets, nanoparticles, or bulk aggregated powders. Morphology engineering mainly optimizes stress-transfer efficiency, maximizes strain-induced dipole oscillation, and tunes stress distribution inside COFs particles. It should be emphasized that morphology regulation cannot create intrinsic molecular dipoles for centrosymmetric building-block systems; it only amplifies the pre-existing piezoresponse rather than generating piezoelectricity from scratch. Therefore, combining morphology control with molecular-level symmetry-breaking design is a promising composite modification route.

**Figure 7:**
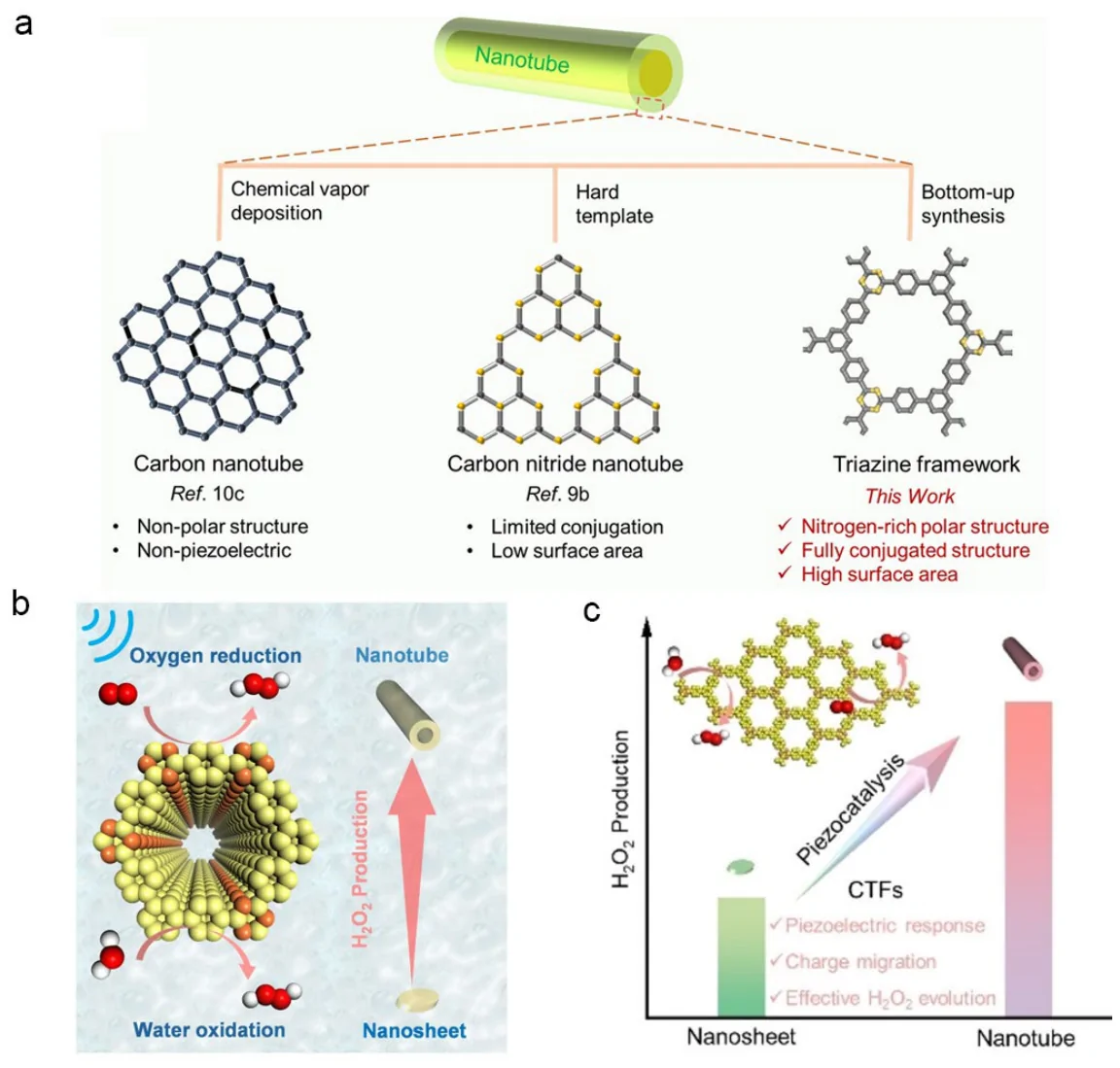
(**a**) Comparison of synthetic strategies and structural features for carbon nanotube, carbon nitride nanotube, and the as-prepared triazine-framework nanotube in this work. (**b**) Schematic illustration of piezocatalytic H_2_O_2_ generation over triazine-framework nanotube via water oxidation and oxygen reduction pathways. (**c**) Schematic comparison of piezocatalytic H_2_O_2_-production performance between CTF nanosheets and nanotubes, highlighting the advantages of nanotube geometry for piezoelectric response, charge migration and H_2_O_2_ evolution (reprinted with permission from Ref. [32]. Copyright 2024, John Wiley & Sons).

Guan and co-workers reported that a one-dimensional tubular covalent triazine nanotube (CTN-1) delivers significantly enhanced piezoresponse in comparison with sheet-shaped CTF-TPB-CN [32]. The PFM response of nanotubular or nanosheet morphologies is significantly stronger than that of bulk powders. This difference arises partly from the enhanced polarization caused by intrinsic stress concentration effects, and partly from changes in tip contact state caused by morphology. It is therefore more suitable as a relative performance reference rather than a quantitative basis for absolute piezoelectric coefficients. Under external pressure, triazine-ring bond-angle deformation produces significant dipoles. CTN-1 achieves outstanding piezocatalytic H_2_O_2_-production rate. Figure 7a compares several representative nanotube-type materials including carbon nanotube, carbon nitride nanotube and the triazine-framework nanotube from this work, highlighting the advantages of nitrogen-rich, fully conjugated triazine tubular structure with high surface area. Figure 7b schematically illustrates piezocatalytic H_2_O_2_ generation over triazine-framework nanotube, where water oxidation and oxygen reduction reactions proceed on nanotube surfaces under mechanical excitation. As quantitatively displayed in Figure 7c, nanotube geometry delivers much higher H_2_O_2_ output relative to its nanosheet counterpart, originating from enhanced piezoelectric response, accelerated charge migration and improved catalytic reaction kinetics within tubular architecture. The tubular geometry can effectively concentrate external stress onto tube walls, promoting bond-angle bending and torsional deformation under mechanical stimuli. Two-dimensional exfoliated COF nanosheets are thinner and prone to bending deformation under fluid shear or ultrasonic agitation; interlayer confinement effect disappears after exfoliation, enabling large-amplitude interlayer relative displacement. By contrast, stacked bulk COF powders suffer from stress dissipation among particle aggregates, and only a limited portion of inner grains can receive effective mechanical strain.

In summary, morphology modulation amplifies pre-existing piezoresponse at micro-nanoscale by optimizing stress distribution and strain-transfer efficiency without changing chemical composition or intrinsic molecular dipoles. Tubular and exfoliated sheet geometries can concentrate external mechanical stress and promote skeleton deformation. A critical limitation is that morphology tuning alone cannot produce piezoelectric response for centrosymmetric COFs building units. The generalized design principle is to combine morphology control with molecular-level symmetry-breaking modification. Optimized micro-nanostructure is utilized to maximize strain delivery toward already-polarizable COFs skeletons, rather than trying to create dipoles from non-polar building blocks.

Morphology modification acts purely as an “amplifier” for pre-existing polarization, which distinguishes it from building-block design and defect engineering that create dipoles at molecular or lattice level. One-dimensional tubular structures achieve prominent stress-concentration effects, yet the synthetic window for COFs nanotubes remains narrow. Exfoliated 2D nanosheets are more widely accessible, but they suffer from easy restacking under liquid-phase catalytic conditions. Morphology optimization cannot compensate for poor intrinsic lattice piezoelectricity. Therefore, it should always be implemented on top of symmetry-broken COFs hosts.

### 3.4. Single-Atom Metal Anchoring

Exogenous metal single atoms are anchored onto COF substrates via coordination bonds to alter local electronic distribution and break local symmetry. Single-atom sites coordinate with heteroatom sites (N, O, S) on COF pore walls, distorting surrounding skeleton bond angles and redistributing electron cloud density, generating extra local dipoles. This strategy simultaneously optimizes piezoresponse and provides catalytic active sites for H_2_O_2_ synthesis and organic-pollutant degradation.

**Figure 8:**
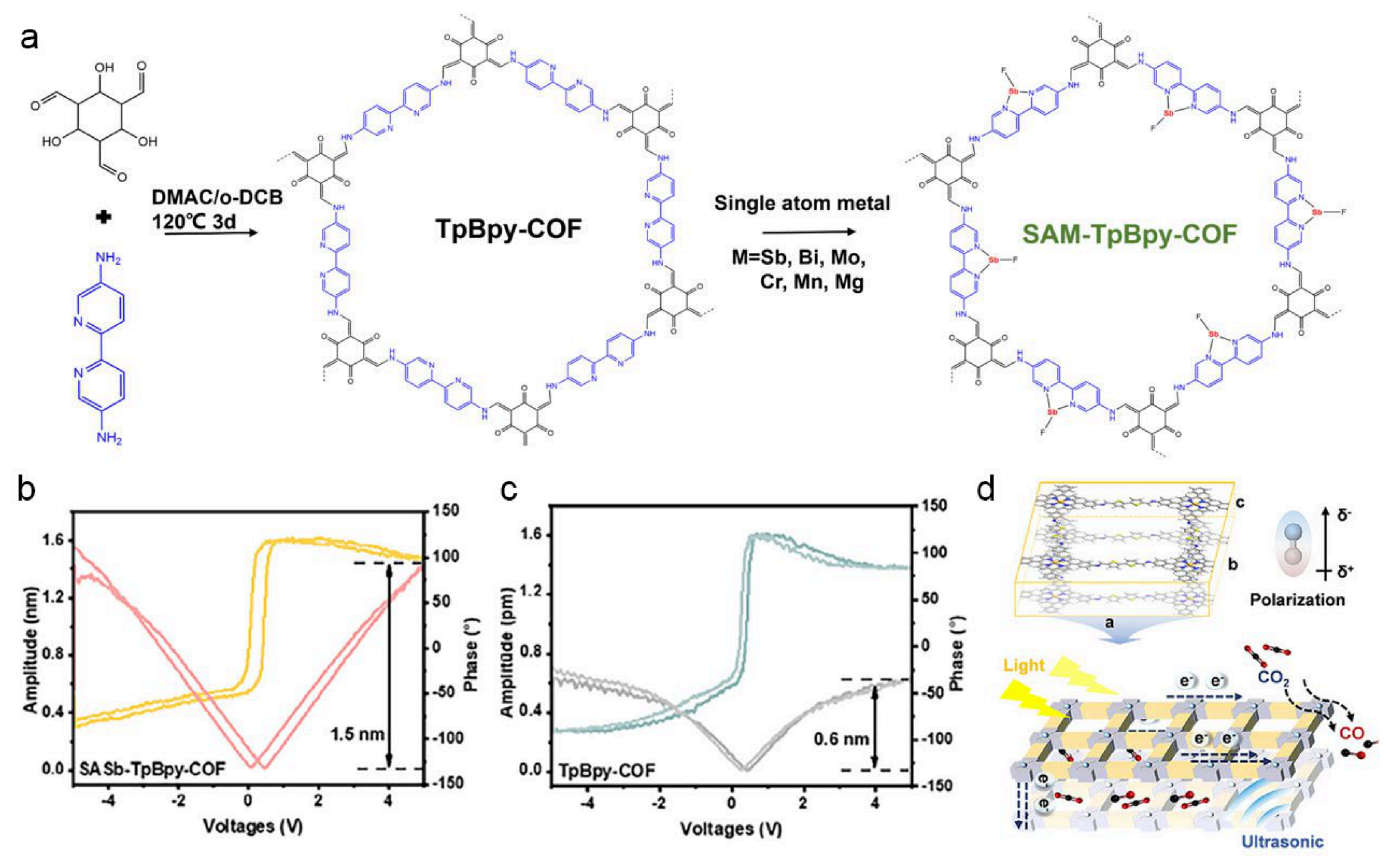
(**a**) Synthetic route for TpBpy-COF and single-atom modified SAM-TpBpy-COF (M = Sb, Bi, Mo, Cr, Mn, Mg). (**b**) PFM characterizations of pristine TpBpy-COF: PFM amplitude-phase hysteresis loops. (**c**) PFM characterizations of SA-Sb-TpBpy-COF: PFM amplitude-phase hysteresis loops (reprinted with permission from Ref. [58]. Copyright 2025, John Wiley & Sons). (**d**) Schematic diagram illustrating polarization-driven charge separation under simultaneous light and ultrasonic excitation for CO_2_ reduction (reprinted with permission from Ref. [59]. Copyright 2025, John Wiley & Sons).

Jiao and co-workers reported that, in SASb-TpBpy-COF, single Sb atoms coordinate with bipyridine subunits via Sb–N orbital hybridization [58]. As depicted in Figure 8a, the synthetic procedure of single-atom modified SAM-TpBpy-COF is realized by implanting diverse metal single atoms onto the TpBpy-COF framework. PFM amplitude-phase hysteresis loops (Figure 8b,c) further confirm enhanced piezoelectric response after Sb single-atom immobilization. PFM signals are enhanced after metal coordination, verifying the generation of local dipoles. The improved PFM signal arises from metal-center intrinsic local polarization and surface-charge-driven electrostatic interactions, to be further confirmed by optical measurements such as second-harmonic generation.

Apart from introducing local dipoles, single-atom anchoring can simultaneously modulate exciton relaxation dynamics and enrich target catalytic active sites. As demonstrated in Zn-Por-COF, single-Zn-atom sites coordinated within porphyrin subunits break local inversion symmetry and establish an intrinsic piezo-internal field. The strain-activated piezoelectric field effectively suppresses phonon-assisted non-radiative recombination and extends carrier lifetime. Benefiting from this synergy, Zn-Por-COF exhibits outstanding piezo-photocatalytic CO_2_ to CO conversion performance of 15.59 mmol g^−1^ h^−1^. Figure 8d illustrates the piezo-photocatalytic CO_2_ to CO reduction mechanism over Zn-Por-COF, where combined light and ultrasonic excitation drive charge separation and CO_2_ conversion [59]. Chen and co-workers reported that dual-single-atom modification offers great potential for further performance optimization [60]. Moreover, the bimetallic Sb-Fe-COF establishes a unique dual-site piezocatalytic mechanism. Specifically, the Sb atomic sites function as piezoelectric electron pumps to accelerate piezocharge generation, while the adjacent Fe sites serve as confined Fenton reaction centers for the in-situ activation of H_2_O_2_. The intrinsic H_2_O_2_ spillover channels in the COFs skeleton facilitate direct intermediate transport and alleviate mass transfer loss.

In summary, single-atom metal anchoring breaks local inversion symmetry at atomic scale via coordination-induced lattice distortion, generating extra local dipoles while concurrently constructing catalytic active sites. This strategy can modulate electronic distribution and accelerate charge-carrier separation. Its practical constraints include limited metal loading capacity and risk of pore-channel blockage at excessive single-atom content. The generalized design principle is to select suitable metal sites to trigger moderate local bond-angle distortion for generating local dipoles, while maintaining high porosity and unobstructed mass-transport pathways inside COFs pore networks. Dual-single-atom systems offer additional opportunities to separate piezopolarization function and catalytic function on independent atomic sites.

Unlike building-block or defect engineering that modifies the whole framework or large lattice regions, single-atom anchoring generates polarization only around isolated metal coordination sites. As a result, the overall macroscopic piezoresponse enhancement is usually moderate. A unique merit is that catalytic active sites and polarization sites can be deliberately decoupled, which cannot be easily realized in twisted-unit or zwitterionic COFs. Nevertheless, distinguishing true piezo-related signals from local electrostatic effects in PFM measurements becomes more complicated for single-atom decorated COFs.

### 3.5. Heterostructure Construction

Constructing multi-component heterointerfaces by coupling COFs with inorganic semiconductors, metal nanoparticles and functional polymers has become a feasible strategy to boost catalytic performance. It is important to distinguish two distinct categories of performance enhancement in such systems: (1) single-factor improvements, which arise from optimization of one individual parameter such as increased specific surface area, enhanced pollutant adsorption, improved interfacial conductivity, optimized band alignment or promoted charge separation; and (2) true synergistic effects, where the overall performance significantly exceeds the arithmetical sum of the performances of each individual component, resulting from the cooperative coupling of multiple functional mechanisms. For most heterostructure systems, both categories of effects coexist, and their relative contributions vary with material design. In addition, the PFM response is significantly improved at the heterostructure interface. The enhanced PFM signal at the interface originates from both the synergistic coupling effect of two-phase polarization and the electrostatic contribution caused by interfacial charge accumulation, which requires cross-validation combined with transient piezoelectric current measurements.

As the most fundamental structural design, ferroelectric perovskite piezoceramics (ZnO, BaTiO_3_, BiFeO_3_ and related phases) are widely integrated with imine-based covalent organic frameworks to construct binary piezo-photocatalytic heterostructures with improved charge-separation efficiency. The performance of these composite systems originates from combined contributions of the inorganic piezoelectric component, heterojunction built-in electric-field effects and interfacial strain-coupling, rather than solely from intrinsic piezoelectricity of the COF lattice. Gao et al. reported a core–shell Z-scheme heterostructure where piezoelectric BaTiO_3_ nanowires were covalently bonded with a 1,3,5-triformylphloroglucinol-p-phenylenediamine-based covalent organic framework (TpPa-1-COF) [61]. By taking advantage of piezopotential and rationally regulated band alignment, the composite overcomes the inherent drawbacks of single-phase materials for photocatalytic water splitting. Benefiting from expanded visible-light absorption, optimized band configuration, fully exposed active sites, and piezo-accelerated charge separation, the covalently connected heterostructure greatly elevates the transfer and utilization efficiency of charge carriers and delivers prominent hydrogen-evolution performance under photo-piezo coupling conditions. This research offers a reliable route for developing high-efficiency catalysts and underscores the great prospect of piezopotential for promoting photoredox reactions. When evaluating composite piezo-photocatalytic heterostructures, one must recognize that performance improvements may originate from two largely independent contributions: conventional heterojunction-driven charge separation and strain-amplified piezoelectric polarization.

**Figure 9:**
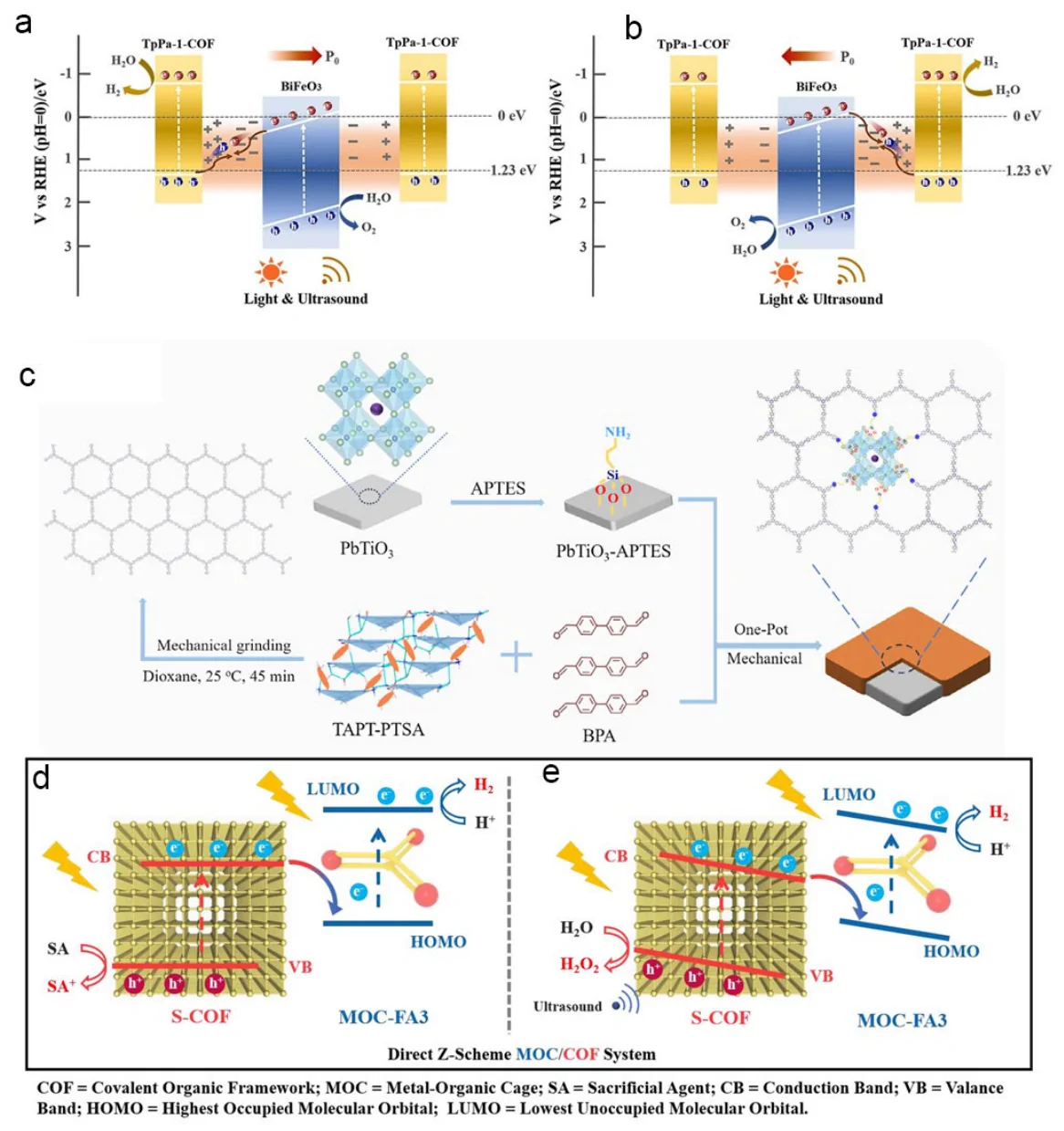
(**a**,**b**) Piezo-photocatalysis with the different direction of polarization for BiFeO_3_@TpPa-1-COF heterojunction (reprinted with permission from Ref. [62]. Copyright 2026, John Wiley & Sons). (**c**) Synthesis and morphology of PTO@COF (reprinted with permission from Ref. [65]. Copyright 2026, John Wiley & Sons). (**d**,**e**) Mechanistic diagram of Z-scheme MOC-FA3/S-COF heterojunction for water splitting: (**d**) Photocatalytic pathway under visible light; (**e**) synergistic piezo-photocatalytic pathway under concurrent ultrasound/light (reprinted with permission from Ref. [68]. Copyright 2026, John Wiley & Sons).

Xu et al. realized, for the first time, a covalent linkage between COFs and piezoelectric materials to fabricate a Z-scheme core@shell heterostructure for overall water splitting [62]. In Figure 9a,b, the band alignment and charge-transfer behaviors of the BiFeO_3_/TpPa-1-COF Z-scheme heterostructure are presented under simultaneous light and ultrasound irradiation. P_0_ represents the built-in electric field originating from piezoelectric polarization. Under the combined excitation of light and ultrasound, the piezoelectric polarization-induced internal electric field modulates the band bending and drives Z-scheme interfacial charge migration. In Figure 9a, photogenerated holes accumulate on BiFeO_3_ and participate in water oxidation to produce O_2_, while electrons on TpPa-1-COF are consumed for H_2_ evolution. By contrast, Figure 9b shows the alternative charge-transfer pathway, where holes from TpPa-1-COF trigger water oxidation and electrons from BiFeO_3_ migrate toward TpPa-1-COF for proton reduction to H_2_. The built-in piezoelectric field facilitates the spatial separation of photogenerated carriers and suppresses undesirable charge recombination in the Z-scheme heterojunction. The optimized BiFeO_3_@TpPa-1-COF photocatalyst achieves remarkable H_2_ and O_2_ evolution rates of 1416.4 and 708.2 μmol g^−1^ h^−1^ under simultaneous ultrasonic excitation and light irradiation, respectively. Following this concept, Huang et al. developed another covalently linked Z-scheme heterostructure combining TpDb-COF and barium titanate (BTO) [63]. The band-alignment diagrams depict the charge-transfer behaviors of the TpDb-COF/BTO heterostructure under individual light irradiation, sole ultrasonic excitation, and coupled light–ultrasound conditions. The switchable ferroelectric piezopotential of BaTiO_3_ modulates interfacial band bending and drives directional carrier migration, which suppresses charge recombination and boosts piezo-photoredox reaction kinetics.

On the basis of basic binary heterojunction construction, core–shell structured ferroelectric@covalent-organic-framework composites realize functional upgrading by introducing surface enrichment effect, which integrates pollutant adsorption and piezo-photocatalysis to solve the problem of insufficient interfacial reaction substrates. In this regard, Wan et al. fabricated a well-defined barium titanate@TD-COF core–shell composite via in situ polycondensation of 1,3,5-tris(4-aminophenyl)benzene and 2,5-dimethoxyterephthalaldehyde on the surface of ferroelectric barium titanate nanoparticles [64]. The porous TD-COF shell possesses prominent adsorption capacity, which can effectively enrich organic pollutants on the material surface and shorten the mass transfer distance for catalytic degradation. Under the synergistic excitation of ultrasound and visible light, the piezoelectric internal electric field originating from lattice dipole reorientation within the barium titanate core accelerates photogenerated charge separation. The perfect combination of adsorption enrichment and piezo-photocatalysis achieves efficient removal of organic pollutants, delivering a novel and high-efficiency water-purification strategy compared with single-component catalytic materials.

To further solve the loose interfacial contact issue in conventional composite systems, researchers have developed covalent-bonded perovskite–imine–COF heterostructures to strengthen interfacial charge coupling. Different from traditional solvothermal methods, Liu et al. adopted a mechanochemical grinding strategy to realize a covalent connection between amine-functionalized lead titanate (PTO) and an imine-based COF [65]. Figure 9c depicts the synthetic strategy of the PbTiO_3_@COF composite, where APTES-functionalized PbTiO_3_ reacts with COF monomers through a one-pot mechanical route. The tightly bonded heterointerface optimizes electronic coupling and facilitates strain transmission across the interface, which significantly enhances the macroscopic piezoelectric response. Benefiting from the stable covalent interfacial interaction, mechanical strain can be efficiently transferred from the piezoceramic to the COF skeleton; the composite exhibits excellent ultrasonic catalytic degradation performance, far superior to physically mixed samples with weak interfacial contact.

Beyond conventional interfacial modification strategies, confined-space ferroelectric@covalent-organic-framework architectures further regulate catalytic reaction pathways by constructing micro-reactor environments. Guo et al. designed a barium titanate@β-COF architecture, where the nanoporous β-COF shell builds closed micro-reactors [66]. The confined microenvironment effectively regulates the transformation of reactive oxygen species, realizing the deep conversion of catalytic intermediates and greatly improving the mineralization efficiency of organic pollutants.

Apart from inorganic–COFs binary systems, metal–organic cage–COFs heterostructures open new avenues for piezo-photocatalysis, where both subunits can deliver intrinsic piezoelectric contributions. Liang et al. fabricated a direct Z-scheme single-atom MOC-Q3/EA-COF piezo-photocatalytic system for overall water splitting [67]. The optimized MOC-Q3/EA-COF delivers a high H_2_ evolution rate of 26.17 mmol g^−1^ h^−1^ together with a Pd-based TON of 118,521 using ascorbic acid as the sacrificial agent. Such outstanding performance originates from its broad light-harvesting capability, efficient charge-carrier separation, and well-dispersed Pd active sites, representing one of the highest activities among COF-based solar-driven H_2_-evolution photocatalysts reported to date.

Building on this concept, Li et al. reported a dual-piezoelectric MOC-FA3/S-COF Z-scheme heterojunction for solar-mechanical water splitting [68]. Distinct from most composite systems where only one component provides a piezoelectric response, both supramolecular MOC-FA3 and S-COF possess non-centrosymmetric crystal configurations. Non-centrosymmetric structural motifs inside MOC-FA3 produce intrinsic molecular dipoles under mechanical stimulation, and S-COF delivers complementary lattice polarization. Supramolecular interactions enable efficient strain coupling across the heterojunction interface, allowing mechanical energy to drive synchronous dipole polarization of both components. The dual piezopotential fields cooperate with Z-scheme charge redistribution to accelerate interfacial charge separation, while well-identified active sites on MOC-FA3 and S-COF separately drive hydrogen evolution and two-electron water oxidation toward hydrogen peroxide production. This work establishes metal–organic cages as a new category of piezoelectric building blocks and demonstrates the merits of dual-piezoelectric heterojunction engineering. Figure 9d,e schematically describe the direct Z-scheme charge transfer of the dual-piezoelectric MOC-FA3/S-COF heterostructure. Under light-only conditions (Figure 9d), the sacrificial agent participates in charge consumption; upon combined light–ultrasound excitation (Figure 9e), piezopotentials originating from both S-COF and MOC-FA3 synergistically accelerate interfacial charge separation, enabling concurrent hydrogen evolution and two-electron water oxidation toward H_2_O_2_ production.

In addition to ferroelectric piezoceramic-framework composites, non-ferroelectric piezoelectric semiconductor@COF heterostructures broaden application boundaries toward practical marine resource exploitation. Qi et al. reported a wave-driven Z-type ZnO@COF heterostructure prepared by in situ growth of covalent organic frameworks on the ZnO surface for piezocatalytic uranium extraction from natural seawater [69]. The outer COF shell stabilizes the inner ZnO, supplies abundant accessible active sites, and establishes rapid electron-transport pathways. The interfacial electric field formed within the Z-scheme heterostructure drives directional electron transfer from the COF toward the ZnO and relieves the edge charge-shielding effect of the ZnO. Heterointerface engineering modulates local lattice distortion of the ZnO and amplifies its intrinsic polarization under mechanical agitation, contributing to outstanding piezocatalytic capability. Relying on mechanical energy harvested from ocean waves, this composite achieves favorable uranium extraction performance in authentic seawater, opening up a promising route for developing piezocatalysts toward marine resource utilization.

Subsequently, all-organic piezoelectric composite systems broaden material application scenarios by virtue of excellent flexibility and processability. Zhang et al. prepared hierarchical porous S-COF/PVDF-HFP composite films through a phase-inversion method [70]. The energy-band evolution of the S-COF/PVDF-HFP composite is depicted. After constructing an organic heterointerface, band bending occurs at the contact region. Under simultaneous light and ultrasonic stimuli, piezoelectric polarization promotes efficient spatial separation of electron–hole pairs across the organic interface, supporting high-efficiency pollutant degradation and antibacterial activity. In one study by Lei and colleagues, functional S-scheme BaBi_4_Ti_4_O_15_@COFs (BBTNC) heterojunction fillers were integrated into PVDF-TrFE (PT) to fabricate piezoelectric-photocatalytic mixed-matrix membranes (BBTNC-MMM) [71]. Compared with the bare PT membrane, the resulting BBTNC-MMM exhibited greatly boosted performance: its piezoelectric output voltage reached 3.8 V and the hydrogen evolution rate was 2072.3 µmol h^−1^ m^−2^, corresponding to >3-fold and 36-fold enhancements, respectively.

On the basis of dual-component design, ternary multi-heterostructures further integrate multiple functional advantages to realize complementary performances and break the performance bottleneck of binary composites. Sun et al. assembled a ternary piezocatalytic system combining piezoelectric NiFe-LDH, highly conductive MXene, and high-porosity COF-LZU1 [72]. Each component undertakes specific functions: the piezoelectric unit provides polarization response and active sites, MXene accelerates interfacial charge transmission, and COF-LZU1 offers abundant surface reaction sites. The synergistic effect of multiple components realizes dual-pathway hydrogen peroxide production and efficient dye degradation. Similarly, Xu et al. constructed Cu-modified ZnO/P-COF ternary composites, where copper doping modulates local lattice distortion and strengthens the intrinsic piezoelectric polarization of zinc oxide, and porous P-COF extends carrier lifetime and expands reaction interfaces [73]. Furthermore, Li et al. built an Ag@Ph-g-C_3_N_4_/P-COF multi-level donor–acceptor ternary architecture with single-atom modification [74]. The synergistic acid-base active sites and piezo-enhanced charge separation realize highly selective piezo-photocatalytic synthesis, fully demonstrating the superiority of multi-component composite design.

With the gradual maturity of structural and functional optimization, piezoelectric framework-based heterostructures have evolved from traditional passive catalysis to intelligent responsive systems, realizing cross-disciplinary functional innovation. Different from conventional environmental catalytic materials, bio-responsive piezoelectric composites achieve precise intelligent regulation through biological-signal activation. Wang et al. designed a pathogen-responsive ZnO@Viologen-COF heterojunction with redox-active viologen moieties embedded within the organic backbone [75]. The specific metabolic electrons of pathogenic bacteria can trigger the redox transformation of the composite material, which breaks the local charge balance and induces lattice distortion that greatly amplifies the piezoelectric performance. Under external photo-ultrasonic coupling stimulation, the intelligent composite significantly improves reactive-oxygen-species generation, realizing selective inhibition of pathogenic bacteria without damaging beneficial microorganisms, which achieves precise in-vivo anti-inflammatory treatment and expands the application of piezoelectric covalent organic frameworks in intelligent biomedicine.

Ultimately, the multi-energy coupling system based on piezoelectric covalent organic frameworks realizes the transformation from laboratory catalytic optimization to practical engineering application, representing the latest development direction of this field. Liu et al. constructed a Por-COF/piezo-aerogel hybrid evaporator for solar-mechanical synergistic seawater desalination [76]. The donor–acceptor structured porphyrin–benzothiadiazole Por-COF optimizes water-cluster formation and reduces water evaporation enthalpy via its ordered porous structure, while the piezoelectric aerogel captures low-frequency mechanical-wave energy to generate an internal electric field, which activates interfacial water molecules and narrows the material band gap. The synergistic photothermal and piezoelectric mechanical-energy utilization achieves high-efficiency seawater evaporation, providing a novel multi-energy coupling strategy for practical seawater-desalination applications based on piezoelectric organic-framework composites. Notably, this hybrid evaporator relies on low-frequency fluid wave agitation rather than ultrasonic excitation, which avoids sonochemical side-effects commonly encountered in piezocatalytic batch tests.

In summary, the contribution of the COFs component in piezoelectric heterostructures is highly system-dependent. In most core–shell or conventional heterostructures, the inorganic phase serves as the dominant piezoelectric source, and the COFs component mainly plays auxiliary roles including adsorption enrichment, mass transport promotion, interfacial charge modulation and strain-transfer optimization. Only in specifically designed dual-piezoelectric systems does the intrinsic piezoelectricity of COFs make a direct and significant contribution to the overall polarization. Distinguishing these different contribution modes is critical for the rational design of high-performance COFs-based piezoelectric heterostructures.

Notably, throughout the structural and functional evolution of these piezoelectric heterostructures, performance improvements stem from both single-factor optimizations and genuine synergistic coupling effects. Adsorption enrichment from porous COF shells, improved interfacial conductivity, and optimized band alignment belong to single-factor improvements, while cooperative coupling between piezoelectric polarization and photoexcitation, or dual piezoelectric polarization from two components, constitute true synergistic effects. Rigorous control experiments with single-component references are essential to quantify the relative contributions of each effect and avoid overstating synergistic effects. Moreover, the interfacial contact mode including covalent bonding, supramolecular adsorption and physical mixing plays a decisive role in interfacial piezocharge-transfer efficiency, since favorable interfacial contact guarantees effective strain delivery for triggering piezopolarization, which provides important guidance for the precise design of high-performance piezoelectric catalysts.

In summary, the five modification strategies introduce symmetry breaking and enhance piezoresponse at different spatial scales, and each modulates the set of key structural parameters (symmetry, dipole orientation, crystallinity, porosity, charge transport, mechanical deformability) in distinct ways, with inherent advantages and limitations.

Building-block molecular design achieves intrinsic symmetry breaking at the molecular scale and does not require post-treatment. It directly tunes molecular dipole magnitude and skeleton flexibility, but it faces the inherent trade-off between dipole density and crystallinity: highly polar or twisted monomers often generate stronger individual dipoles, but hinder long-range ordered packing, leading to reduced macroscopic polarization. The optimal performance usually appears at a moderate symmetry-breaking level that balances dipole strength and crystallinity.

Defect engineering introduces local polarization at the lattice scale with mild impact on overall framework crystallinity. Defect sites break local inversion symmetry and generate additional dipoles, and they also serve as charge trap states that moderate carrier migration. Moderate defect density enhances both polarization and surface catalytic activity, but excessive defects destroy long-range order, reduce charge mobility and weaken structural stability.

Morphology modulation amplifies pre-existing piezoresponse at the micro-nanoscale by optimizing stress distribution and transfer efficiency without changing the chemical composition. Tubular or sheet-like morphologies concentrate mechanical stress and promote skeleton deformation, but they also change specific surface area and mass transport properties. Notably, morphology regulation cannot generate piezoelectricity from centrosymmetric building units alone; it must be combined with molecular-level symmetry breaking to be effective.

Single-atom anchoring introduces local dipoles and catalytic active sites simultaneously at the atomic scale. The coordinated metal centers distort surrounding bond angles to break local symmetry, and they also modulate electronic structure to facilitate charge transfer. However, the loading amount and dispersion uniformity are technically limited, and excessive single-atom loading may block pore channels and reduce porosity.

Heterostructure construction achieves synergistic polarization enhancement at the interface scale and integrates multiple functionalities. The built-in interfacial electric field promotes charge separation and extends carrier lifetime, but interfacial strain-transfer efficiency and contact quality become critical performance-limiting factors. Poor interfacial bonding not only hinders charge transfer across the interface, but also blocks stress transmission from one component to the other, greatly weakening the piezoelectric synergistic effect.

Combinations of multiple strategies (e.g., molecular design + morphology control + heterostructure construction) represent a promising direction to achieve further performance breakthroughs, as they can compensate for the limitations of individual strategies through multi-scale synergistic modulation.

From a cross-strategy comparative standpoint, intrinsic symmetry breaking via building-block design produces the most straightforward lattice-originated piezoresponse, yet synthetic constraints on crystallinity are severe. Defect and single-atom routes create only local polarization, which is hard to quantify experimentally. Morphology tuning cannot create dipoles, but can effectively boost existing performance. For heterostructures, performance improvement frequently mixes contributions from classical heterojunction charge separation and true piezo-coupling effects, making effect deconvolution a major experimental challenge.

## 4. Applications of Piezoelectric COFs

Benefiting from the combined advantages of piezoelectric polarization, permanent porosity, excellent chemical stability, and tunable electronic structure, COF-based piezo-(photo)catalysts have been explored in fields including clean-energy chemical conversion, environmental remediation, heavy-metal ion removal, CO_2_ reduction, antibacterial disinfection, and mechanical-energy collection, which are summarized in Table 2.

### 4.1. Piezocatalytic and Piezo-Photocatalytic Water Conversion

Water conversion, including water splitting for hydrogen evolution and H_2_O_2_ photosynthesis, is a core energy-oriented application. Piezocatalysis can realize water conversion under dark vibration conditions, while piezo-photocatalysis achieves higher efficiency via light-force synergy.

Beyond conventional twisted and D–A-type COF catalysts, zwitterionic SQ-TAPT provides a new paradigm for piezocatalytic hydrogen evolution driven by ionic-displacement polarization [56]. Benefiting from large Born effective charges inside the zwitterionic backbone, SQ-TAPT builds a strong built-in electric field upon mechanical perturbation, which facilitates the spatial separation of piezogenerated charge carriers. Multiple characterizations collectively confirm its superior charge-separation and charge-transport capability compared with isostructural neutral PDA-TAPT. Zwitterionic SQ-TAPT delivers a piezocatalytic H_2_-production rate of 745.9 μmol g^−1^ h^−1^ under dark ultrasonic conditions, which is 2.92-fold higher than the neutral isostructural PDA-TAPT, directly verifying the advantage of ionic-displacement polarization originating from zwitterionic skeletons. Its improved surface hydrophilicity and optimized hydrogen-adsorption behavior jointly lower the kinetic barrier of the hydrogen-evolution reaction. Under dark ultrasonic vibration, co-catalyst-free SQ-TAPT reaches competitive piezocatalytic hydrogen-production performance and maintains stable activity after cyclic tests, exhibiting competitive performance among reported piezocatalytic hydrogen-production catalysts. This work highlights that engineering backbone charge distribution to trigger atomic-level ionic-displacement polarization is an effective molecular-level strategy to advance COF-based piezocatalytic water splitting.

**Figure 10:**
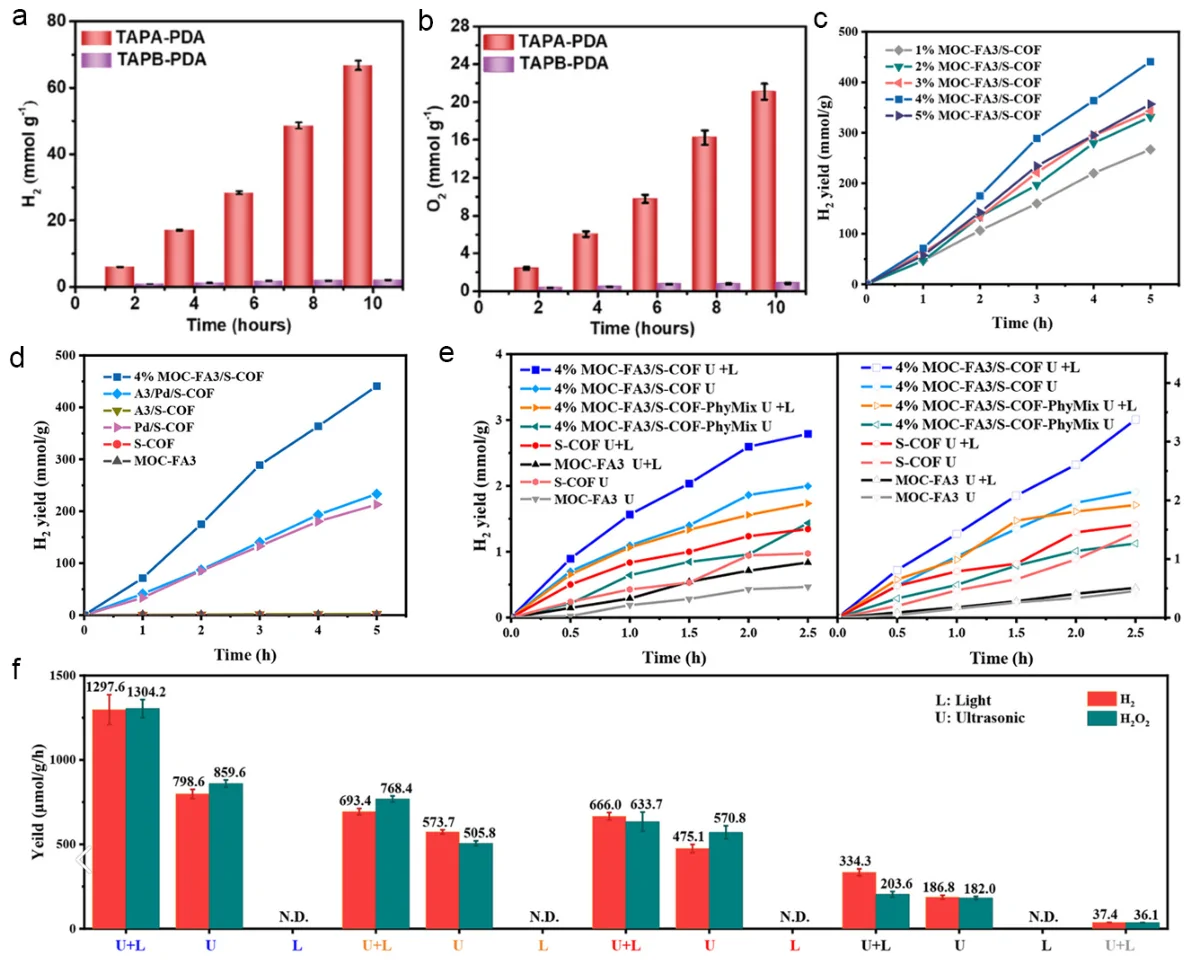
Catalytic performance evaluation of piezo-photocatalytic/piezocatalytic water conversion systems. (**a**) Time-dependent H_2_ evolution and (**b**) O_2_ evolution TAPA-PDA and TAPB-PDA COF samples under 200 W, 40 kHz ultrasonic excitation without light irradiation (reprinted with permission from Ref. [54]. Copyright 2026, John Wiley & Sons). (**c**) Time-dependent H_2_-evolution yields for MOC-FA3/S-COF composites with different MOC-FA3 loading fractions under visible-light irradiation (λ > 420 nm, Xe lamp). (**d**) Comparative time-dependent H_2_-evolution yields of various reference materials under identical visible-light conditions (λ > 420 nm, Xe lamp, 5 h). (**e**) H_2_-production yields of MOC-FA3, S-COF, physical-mixed 4% MOC-FA3/S-COF-PhyMix, and integrated 4% MOC-FA3/S-COF heterostructure under ultrasound-only (U), light-only (L), and combined light–ultrasound (U + L) excitation. (**f**) Quantitative bar-chart comparison of H_2_ and H_2_O_2_ yields over diverse catalysts under different excitation modes; N.D., not detected. Figure 10c–f are reprinted with permission from Ref. [68]. Copyright 2026, John Wiley & Sons.

In addition, TAPA-PDA represents a metal-free ferroelectric COF prototype, whose piezoresponse originates from intrinsic piezopolarization/D–A phonon–dipole coupling, where photogenerated carriers accumulate on pore surfaces to drive water-splitting reactions without any metallic catalytic sites [54]. In Figure 10a,b, the time-dependent H_2_ and O_2_ evolution performances of TAPA-PDA and TAPB-PDA COFs are compared under piezo-photocatalytic conditions. TAPA-PDA delivers remarkable H_2_ and O_2_ production over a 10 h reaction, whereas nearly negligible gas evolution is observed for TAPB-PDA, revealing the critical influence of molecular structure on catalytic activity. Great advances in heterostructure engineering have significantly boosted the performance of hydrogen evolution. The COF-TpDb@BTO covalent Z-scheme composite also realizes prominent piezo-photocatalytic hydrogen evolution performance [63].

In addition, under piezo-photocatalytic conditions, COF-DH-Eth delivers a prominent H_2_O_2_-production rate, demonstrating obvious synergistic enhancement between piezoelectric polarization and photoexcitation [52]. COF-DH-Eth achieves a much higher H_2_O_2_ concentration under piezo-photo coupled excitation compared with the sole-light condition. In comparison, COF-DH-H shows relatively limited performance under identical testing conditions, manifesting that skeleton modification further amplifies the piezo-photocatalytic synergistic effect.

In addition, the dual-piezoelectric MOC-FA3/S-COF Z-scheme supramolecular heterostructure achieves co-production of hydrogen and hydrogen peroxide under light–ultrasound co-excitation in pure water [68]. Figure 10c presents the hydrogen-evolution yield over MOC-FA3/S-COF composites with varied MOC-FA3 loading amounts. The 4% MOC-FA3/S-COF sample achieves the highest H_2_ yield, and excess MOC-FA3 (5%) leads to decreased catalytic performance. Figure 10d further compares the catalytic activity among different reference samples. The optimized 4% MOC-FA3/S-COF exhibits far superior H_2_ production compared with single-component S-COF, MOC-FA3, and other control composites. Figure 10e displays the piezo-photocatalytic H_2_ yields under different excitation modes (ultrasound only, light only, ultrasound plus light). The 4% MOC-FA3/S-COF sample shows the largest hydrogen yield under simultaneous ultrasound and light irradiation, and the physically mixed counterpart delivers inferior activity, confirming the necessity of an intimate heterojunction interface. Figure 10f quantitatively summarizes the H_2_ and H_2_O_2_ yields of various catalysts under ultrasonic-light coupled, single-ultrasonic, and single-light excitation. Notably, light-only groups show no detectable product (N.D.), demonstrating that light irradiation alone cannot drive the catalytic reaction. The highest H_2_ and H_2_O_2_ outputs are obtained over 4% MOC-FA3/S-COF under co-excitation of ultrasound and light.

For piezocatalytic H_2_O_2_ synthesis, Li and co-workers developed a piezoelectric catalyst where barium titanate is in situ confined within covalent-organic-framework layers. This BaTiO_3_@COF catalyst featuring dual micro-environments supports two-route piezocatalytic H_2_O_2_ formation, with no sacrificial reagents or extraneous oxygen input needed. Subjected to ultrasonic perturbation, the dual micro-active sites drive the concurrent transformation of water and oxygen into H_2_O_2_. The cooperative reaction mechanism is primarily governed by two-electron water oxidation, with two-electron oxygen reduction acting as the secondary contribution. Furthermore, the unfavorable four-electron water-oxidation pathway is restrained, yielding a competitive H_2_O_2_-production rate of 240.7 µmol g^−1^ h^−1^ [77]. As documented by Sun and co-workers, the ternary NiFe-LDH/MXene/COF-LZU1 heterostructure can realize effective piezocatalytic H_2_O_2_ generation by triggering water oxidation and oxygen reduction simultaneously. Serving as the central component, NiFe-LDH is celebrated for its prominent catalytic performance. Its characteristic layered geometry endows it with desirable intrinsic activity, facilitating its efficient involvement in catalytic processes. Highly conductive MXene is embedded into the system as an essential structural mediator to boost the rate of internal charge migration. In addition, high-surface-area COF-LZU1 is incorporated, contributing to more exposed active centers and further ameliorating the conductivity of the hybrid material. Collectively, the resultant NiFe-LDH/MXene/COF-LZU1 heterostructure integrates enlarged specific surface area and optimized electron-transport property. It delivers a competitive piezocatalytic H_2_O_2_-production rate of 847.492 μM g^−1^ h^−1^ Impressively, only a 21.8% activity loss is observed upon three successive days of cyclic piezocatalytic assessments [72]. In addition, Bottom-up synthesized piezoelectric covalent triazine-based nanotube CTN-1 features a fully conjugated nitrogen-rich tubular architecture [32]. The prominent nanocurvature effect originating from one-dimensional hollow geometry gives rise to outstanding piezoresponse and facilitates carrier separation. Under test conditions employing pure water and ambient air and free of sacrificial agents or metal-based cocatalysts, CTN-1 achieves an outstanding piezocatalytic H_2_O_2_ generation rate of up to 4115 μmol g^−1^ h^−1^. In sharp comparison, CTF-TPB-CN only attains a piezocatalytic H_2_O_2_ yield of 1560 μmol g^−1^ h^−1^, representing substantially poorer catalytic performance relative to CTN-1, unambiguously revealing the morphology-dependent piezocatalytic superiority of the tubular architecture.

Notably, most reported catalytic performances are obtained under ultrasonic excitation, and contributions from sonochemical side-effects should be ruled out via standardized control experiments as described in Section 2.1.1. Practical performance under weak, low-frequency mechanical stimuli still needs further exploration.

Comparatively speaking, twisted-backbone COFs exhibit outstanding piezo-photocatalytic H_2_O_2_ generation activity, while zwitterionic COFs stand out for dark piezocatalytic H_2_ evolution without metal cocatalysts. 1D tubular-morphology COFs achieve high piezocatalytic rates originating from stress-concentration effects, but such nanotube structures remain difficult to synthesize at large scale. Heterostructure systems can further elevate activity by combining piezopotential and heterojunction charge separation, yet their performance improvement is often entangled with contributions from conventional heterojunction effects, making it hard to isolate pure piezoelectric contributions. At present, direct quantitative comparison across literature reports is hampered by divergent testing conditions (ultrasonic power, catalyst dosage, reactor geometry). Community-wide standardized testing protocols are urgently required to rank material performance fairly.

### 4.2. Piezo-Photocatalytic CO_2_ Reduction for Artificial Photosynthesis

**Figure 11:**
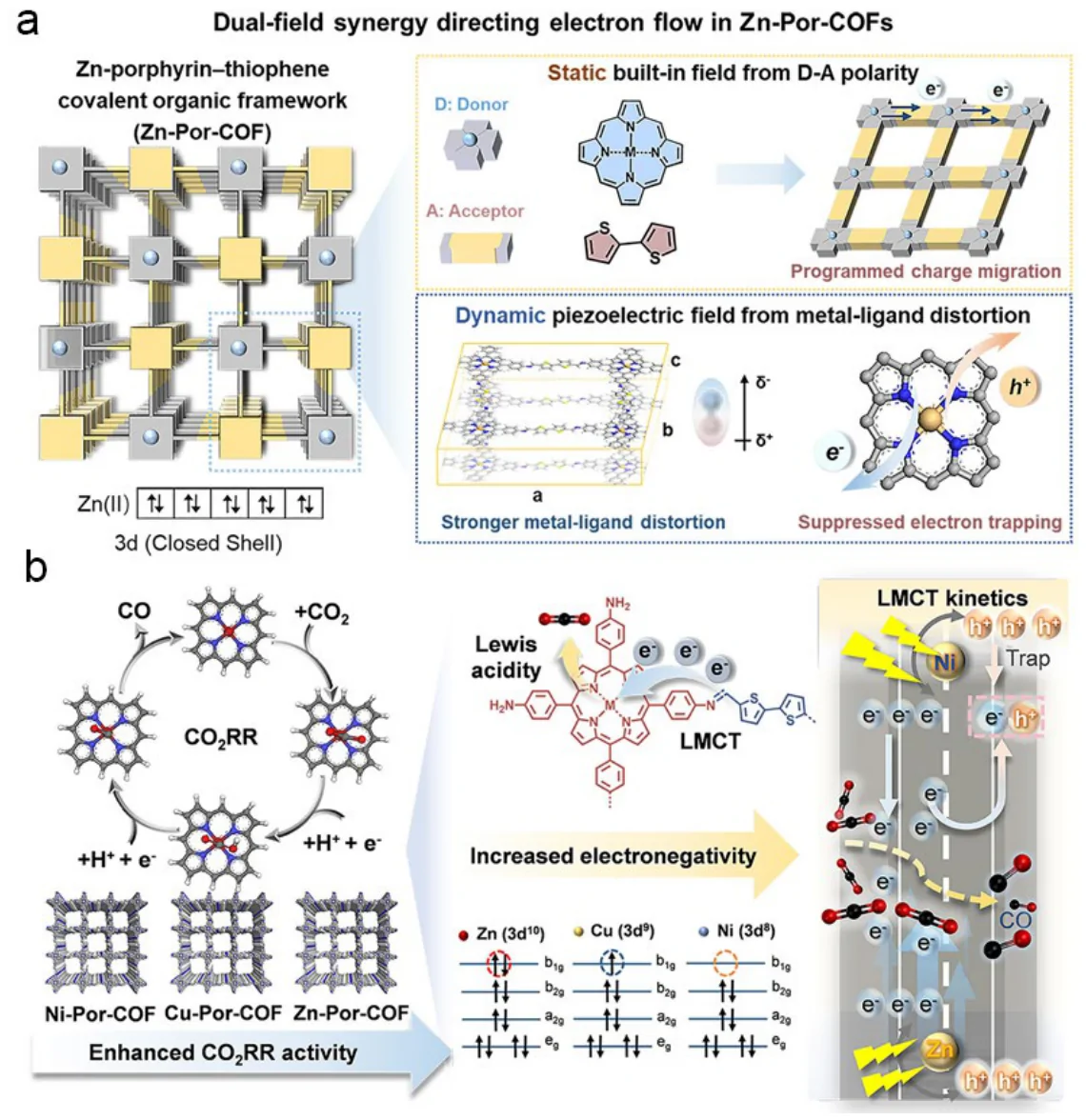
(**a**) Illustration of the dual-field (static and dynamic) modulation mechanism in Zn-Por-COF; (**b**) schematic illustration of the electronic structure and charge transfer mechanisms in M-Por-COF (reprinted with permission from Ref. [59]. Copyright 2026, John Wiley & Sons).

Besides water-based energy conversion, piezo-photocatalytic CO_2_ reduction emerges as a fast-growing application branch for artificial photosynthesis [59]. Zn-Por-COF with single-atom-induced intrinsic piezoelectricity delivers high CO yield of 15.59 mmol g^−1^ h^−1^ under combined light–ultrasound excitation. The time-dependent CO-production yields of a series of Por-COF samples are shown under sole photocatalysis and piezo-photocatalysis conditions, respectively. Obvious activity enhancement can be observed for Zn-Por-COF upon the introduction of ultrasonic vibration. Zn-Por-COF exhibits an optimal CO generation rate under piezo-photocatalytic co-excitation among Zn-Por-COF, Cu-Por-COF, Ni-Por-COF, and bare Por-COF, which demonstrates the prominent synergistic effect between piezoelectric polarization and photoexcitation (Figure 11a). The underlying structure–activity origin and charge-transfer mechanism are illustrated in Figure 11b, where metal-site-modulated ligand-to-metal charge-transfer (LMCT) kinetics and electronic-structure regulation collectively promote the CO_2_RR process.

Compared with piezocatalytic water-splitting systems, CO_2_ photoreduction over piezoelectric COFs remains far less explored. At present, only a handful of intrinsic piezoelectric COFs platforms have been demonstrated for this target reaction, leaving enormous room for material innovation.

### 4.3. Organic-Pollutant Degradation

**Figure 12:**
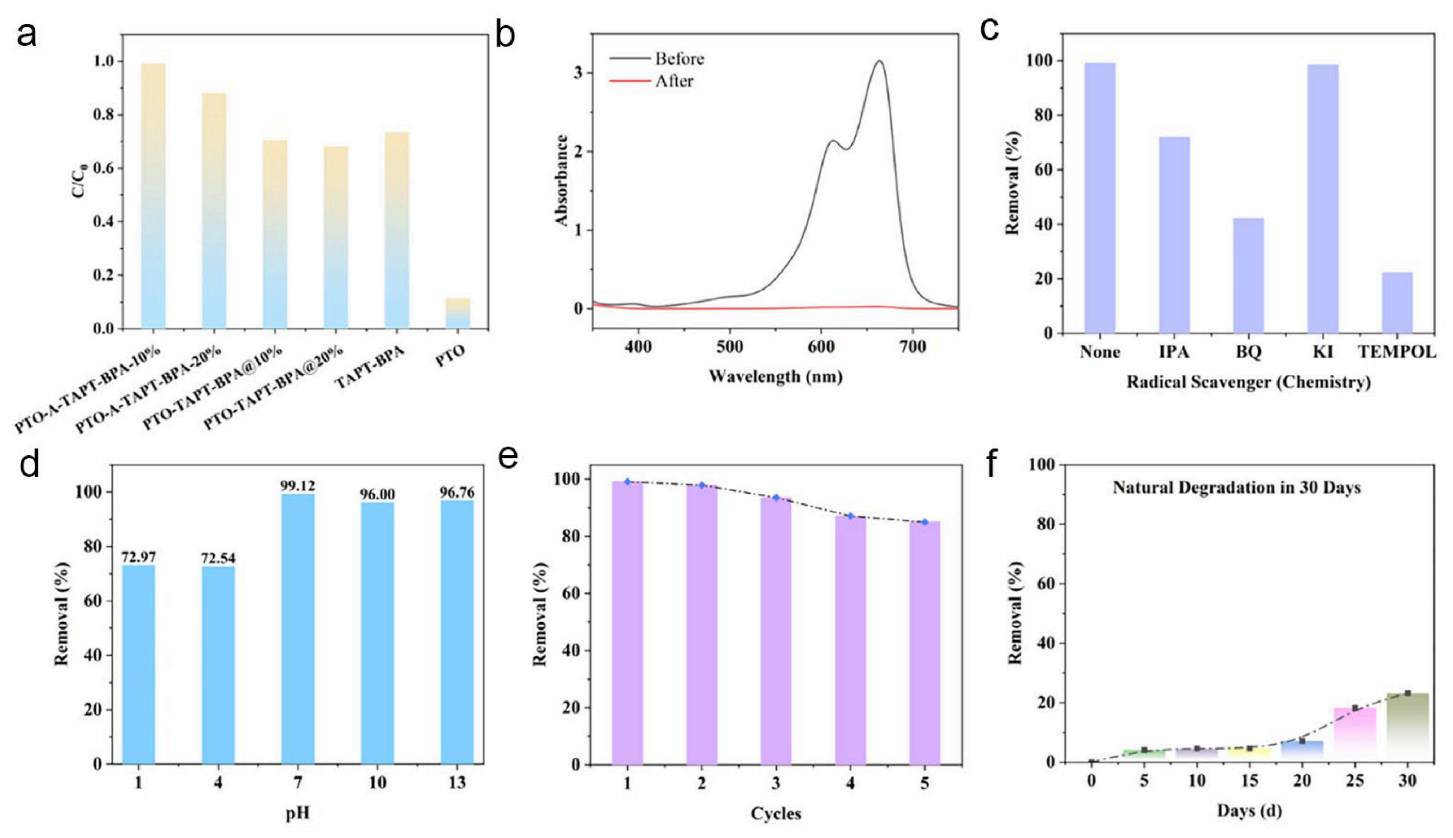
Mineralization of MB. (**a**) Comparison of degradation efficiency of different materials; (**b**) UV-vis absorption spectra of MB solution before and after catalytic degradation by PTO@COF; (**c**) degradation efficiency of MB in the presence of different free radical scavengers (IPA,BQ, KI, TEMPOL), with “None” being the control group without scavengers; (**d**) degradation efficiency of MB by PTO@COF under different pH environments; (**e**) cycle stability test of PTO@COF catalyzed MB degradation (5 cycles); (**f**) natural degradation efficiency curve of MB within 30 days (reprinted with permission from Ref. [65]. Copyright 2026, John Wiley & Sons.

Piezocatalysis has emerged as a promising green catalytic technology that can effectively degrade organic contaminants in water environments relying on mechanical energy without the requirement of light illumination. Guo et al. fabricated covalently bonded PTO@COF heterojunctions via a mechanochemical strategy [65]. The optimized composite achieves nearly complete degradation of methylene blue and retains excellent catalytic performance even in high-salinity complex wastewater systems. As illustrated in Figure 12a, the normalized residual concentration of the target pollutant is compared across various PTO-A-TAPT-BPA composites with different loading ratios, together with TAPT-BPA and bare PTO references. The composite samples exhibit substantially lower residual pollutant concentrations than pure PTO, demonstrating the superior piezocatalytic degradation capability of the heterostructure. Figure 12b displays the UV-vis absorption spectra of the pollutant solution before and after the piezocatalytic reaction. The nearly vanished characteristic absorption peak confirms the efficient elimination of pollutant molecules. Figure 12c presents radical-trapping experiments using different quenchers. The degradation efficiency is drastically suppressed upon the addition of BQ and TEMPOL, implying that superoxide radicals and singlet oxygen serve as the dominant reactive oxygen species for piezocatalytic degradation. By contrast, IPA and KI induce a relatively moderate activity decline. Figure 12d shows the pH-dependent degradation performance. High removal efficiency is achieved across a broad pH window, and optimal degradation activity is obtained under neutral to weak alkaline conditions (pH 7–13). Figure 12e depicts five successive recycling tests. Only slight performance decay is observed after five cycles, verifying the robust reusability of the as-prepared piezocatalyst. Figure 12f evaluates natural degradation within 30 days. Negligible pollutant removal occurs in the blank natural-degradation group, which excludes the interference from spontaneous pollutant decomposition and validates that the observed degradation originates from piezocatalytic processes. Chen et al. developed an Sb-Fe-COF dual-single-atom catalyst to construct a piezo-self-Fenton system [60]. In this architecture, Sb sites act as a piezo-electron pump while neighboring Fe sites function as confined Fenton reactors, jointly enabling efficient degradation of sulfamethoxazole. Lu and co-workers combined piezoelectric BTO with TD-COF to construct a BTO-3@TD-COF heterojunction [64]. The BPA degradation kinetic curves under independent photocatalysis, piezocatalysis, and piezo-photocatalysis demonstrate that BTO-3@TD-COF exhibits far superior degradation capability relative to bare BTO and TD-COF. The corresponding apparent rate constants quantitatively confirm the remarkable synergistic effect originating from the coupling of piezoelectric polarization and photoexcitation. Furthermore, ligand-tuned Bi_2_WO_6_/R-COF (R = H, F, CH_3_O) heterojunctions were developed for SMX elimination [78]. The piezo-photocatalytic degradation mechanism over Bi_2_WO_6_/R-COFs reveals that ultrasound-induced piezoelectric fields facilitate charge separation, and heterojunction interfaces offer abundant channels for reactive oxygen species generation. The comparison of SMX removal efficiency and electron-transfer kinetic constants highlights that appropriate ligand modification can effectively optimize charge distribution and catalytic activity. For organic pollutant degradation applications, pure intrinsic piezoelectric COFs often deliver moderate degradation rates. Most high-efficiency systems reported so far rely on inorganic-COFs heterostructures. It should be noted that pollutant degradation efficiency is strongly affected by initial pollutant concentration, pH, and water-matrix components. Therefore, performance numbers from different publications cannot be simply compared without identical reaction conditions. Dual-single-atom COFs systems provide an interesting alternative route without inorganic phases, but their library is still very limited.

### 4.4. Antibacterial Disinfection

Beyond general non-selective disinfection, emerging bio-responsive piezo-photocatalytic systems achieve pathogen-selective microbiome regulation. Wang et al. developed a redox-switchable ZnO@Viologen-COF (ZVP) heterojunction that specifically responds to respiratory electrons released by pathogenic Cutibacterium acnes [75]. Pathogen-induced viologen reduction greatly boosts piezo-photocatalytic ROS production, realizing targeted inhibition of pathogenic bacteria without damaging beneficial commensal strains, which provides a new perspective for intelligent skin microbiome intervention. In addition, Zhang et al. demonstrated that, driven by strong interfacial hydrogen-bonding interactions, COF nanoparticles with an optimal loading of 5 wt% act as efficient nucleating agents [70]. These nanoparticles remarkably increase the electroactive β-phase fraction within the PVDF-HFP matrix and strengthen its piezoelectric response. Under simultaneous light irradiation and ultrasonic excitation, the optimized composite exhibits robust broad-spectrum antibacterial efficacy against Escherichia coli and Staphylococcus aureus, achieving nearly 99% bacterial inactivation.

Current antibacterial studies based on piezoelectric COFs can be broadly divided into two categories: pathogen-selective bio-responsive systems and non-selective broad-spectrum composite films. Bio-responsive designs achieve targeted microbial inhibition by responding to metabolic signals from pathogenic bacteria, which helps to spare beneficial commensal microorganisms. By contrast, non-selective composite films deliver high-efficiency broad-range disinfection, yet they cannot distinguish harmful bacteria from favorable microbes. Despite these promising laboratory-scale results, real-world practical performance validation under complex biological matrices such as body fluids or actual environmental water samples remains scarce, which hinders further translation toward real-life application scenarios.

### 4.5. Piezocatalytic Uranium (VI) Reduction

Radioactive uranium-containing wastewater poses a severe hazard to the ecological environment and human health. Piezocatalysis offers a new strategy for uranium removal without light irradiation. The D-COF@ZnO heterostructure displays excellent piezocatalytic U(VI) reduction performance under ultrasonic vibration. Piezopotential drives the reduction of U(VI) into insoluble U(IV) [57]. The material maintains high selectivity toward uranium in real wastewater, offering new insights into radioactive wastewater treatment. In this process, piezogenerated electrons reduce soluble uranyl U(VI) to low-valent insoluble U(IV) precipitates anchored on the COF surface. High-valence holes are quenched by sacrificial agents. Selectivity against co-existing competing metal ions is vital for practical application. Defect sites in the COF can serve as uranium adsorption sites, synergistically cooperating with the piezocatalytic reduction process.

Beyond industrial radioactive wastewater remediation, piezocatalysis also enables ocean mechanical energy harvesting for sustainable uranium extraction from natural seawater. As reported by Qiu et al., piezoelectric catalysis holds great potential for converting ubiquitous ocean wave energy into efficient seawater uranium extraction [69]. The synthetic route of the Z-scheme ZnO@COF composite is illustrated. ZnO microspheres are first modified by APTES, followed by in situ solvothermal growth of a TP-BDOH COF shell on the ZnO surface to obtain the target ZnO@COF heterostructure. The outer porous COF shell stabilizes the inner ZnO substrate, provides abundant accessible active sites, and constructs rapid electron-transport pathways. The interfacial electric field formed in the Z-scheme heterojunction drives directional electron transfer from COF to ZnO and alleviates the edge charge-shielding effect of ZnO. Upon ultrasonic wave excitation, piezoelectric polarization takes place within ZnO, generating large amounts of charge carriers. The built-in heterojunction electric field further promotes spatial separation of piezogenerated electrons and holes. The migrated electrons are capable of reducing soluble U(VI) into insoluble U(IV). Moreover, heterointerface engineering effectively amplifies the intrinsic lattice polarization of ZnO, further boosting the macroscopic piezoelectric response and charge-separation efficiency. Benefiting from this synergistic structural and electronic modulation, the optimized ZnO@COF composite achieves an ultrahigh uranium extraction efficiency of 7.56 mg g^−1^ d^−1^ driven by natural ocean wave vibration. This work provides a feasible paradigm for designing high-efficiency piezocatalysts for practical marine uranium resource utilization.

Most of the currently reported high-performance piezocatalytic U(VI) removal examples adopt heterostructure architectures integrating COFs and inorganic piezoelectric components. Such composite architectures can combine the strong piezoresponse of inorganic phases with the favorable adsorption capability and porous skeleton of COF materials. Nevertheless, their practical performance is highly contingent on robust interfacial bonding to ensure efficient strain transfer across heterointerfaces, and inadequate interfacial contact may weaken piezopolarization-related coupling effects. By comparison, piezocatalytic uranium extraction based on intrinsic piezoelectric pure-COF frameworks has not yet been well established, which opens up opportunities for follow-up material exploration.

### 4.6. Piezoelectric Nanogenerators (PENGs) for Mechanical-Energy Harvesting

**Figure 13:**
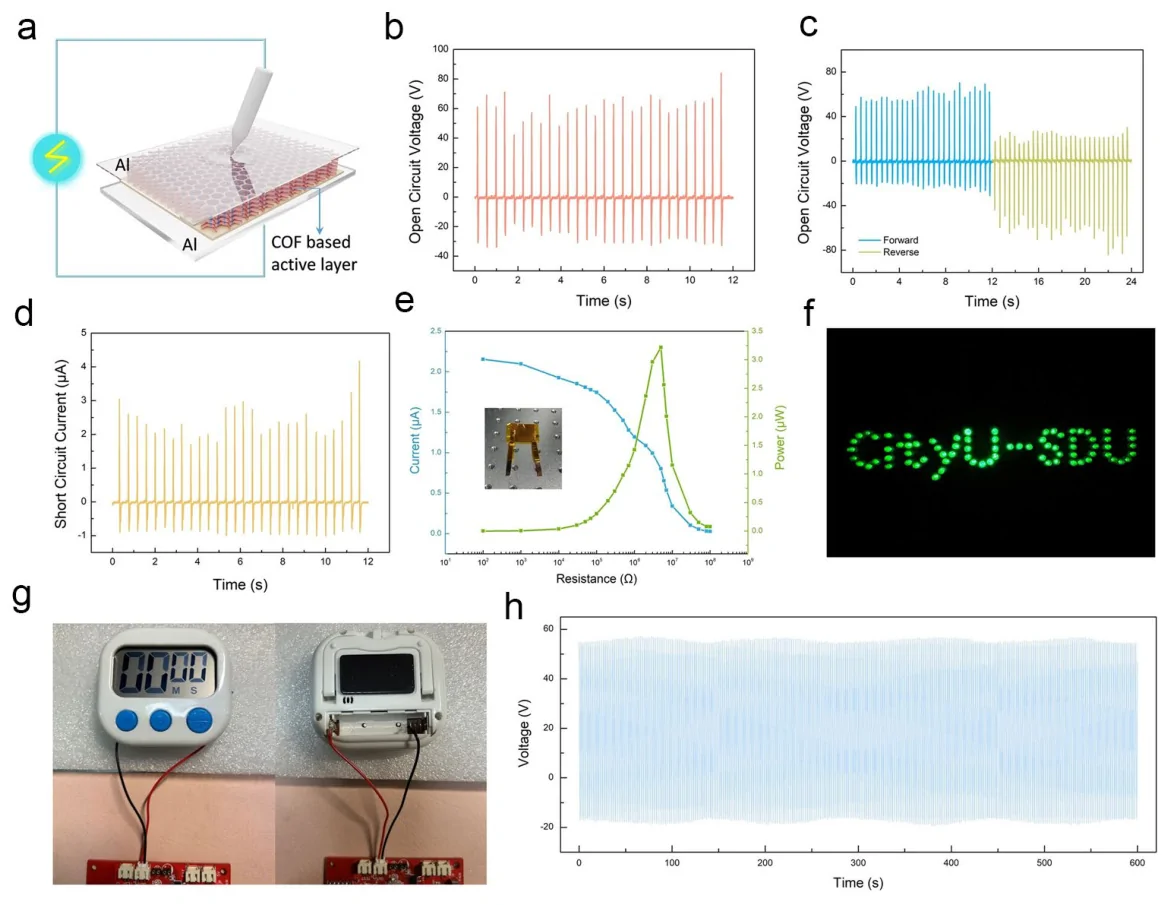
(**a**) Schematic diagram of a piezoelectric energy harvester with Al/active layer/Al architecture. (**b**) Output open-circuit voltage (Voc) of PENG based on CityU-13. (**c**) Voc of PENG based on CityU-13 with both the forward and reverse connections. (**d**) Output short circuit current (Isc) of CityU-13 based PENG. (**e**) Variation of Isc and power with load resistance for PENG based on CityU-13. The inset shows the photo of PENG. (**f**) Photograph of CityU-13 based PENG successfully driving 76 commercial light emitting diodes (LEDs) showing term “CityU-SDU.” (**g**) Photograph of CityU-13 based PENG successfully driving the LED display timer. (**h**) Stability tests over time up to 600 s for PENG based on CityU-13 (reprinted with permission from Ref. [53]. Copyright 2024, John Wiley & Sons).

Piezoelectric COFs are mixed with a PDMS or polymer matrix to fabricate flexible PENG devices, converting mechanical energy into electrical energy for self-powered electronics. Han and co-workers constructed piezoelectric COFs using twisted molecular skeletons. PENG devices were assembled by embedding these piezoelectric COFs between two silver electrodes. Upon 20 N mechanical loading, COF-DH-H and COF-DH-Eth-based PENGs yielded open-circuit voltages of 2 V and 6 V, respectively [52]. Their performance exceeds that of a wide range of organic piezoelectric materials [79,80,81]. A COF-based piezoelectric nanogenerator (PENG) with Al electrodes and a COF active layer was subsequently reported by Zhang and co-workers. Its structural schematic is shown in Figure 13a [53]. Typical electrical output performances of the PENG are presented in Figure 13b–d, where stable open-circuit voltage and short-circuit current can be harvested under mechanical triggering. Forward and reverse connection tests in Figure 13c further verify that the generated electrical signals originate from the intrinsic piezoelectric effect. As shown in Figure 13e, the output current and power of the device vary with external load resistance, demonstrating its practical energy-harvesting capability. Benefiting from the reliable piezoelectric output, such PENG devices can drive LED arrays displaying “CityU-SDU” (Figure 13f) and power commercial electronic timers (Figure 13g). Long-term cyclic voltage output in Figure 13h confirms the robust working stability of the COF-based PENG. COF-based PENGs exhibit application potential for self-powered sensors, wearable electronics, and Internet-of-Things terminals.

In composite PENG films, COF filler is dispersed inside an insulating polymer matrix. The dispersion quality, filler loading fraction, film thickness, and mechanical flexibility jointly determine the output electrical performance. Large-pore COFs may introduce voids inside the composite membrane, which can influence dielectric properties and mechanical durability. Compared with inorganic piezoelectric ceramic fillers, COF–polymer composites possess better flexibility and lower density, yet the current output magnitude still needs further improvement.

Across existing studies, piezoelectric-COF-based piezocatalysis and piezo-photocatalysis exhibit diverse application prospects, including energy-targeted chemical synthesis, CO_2_ artificial photosynthesis, pollutant abatement, targeted antimicrobial therapy, heavy-metal remediation and solid-state mechanical-energy harvesting. Their practical performance is governed by three interrelated factors: intrinsic lattice polarization from symmetry-broken skeletons, improved mass transport via permanent pore networks, and efficient separation of mechanically or photogenerated charge carriers. Pure intrinsic piezoelectric COFs are favorable for dark piezocatalysis under mechanical excitation, whereas composite heterostructures broaden performance boundaries by integrating COFs’ tunable porous features with complementary functions of inorganic phases, metal–organic cages or polymer matrices. As a promising subclass, dual-piezoelectric heterostructures produce piezopotential from both components for accelerated charge separation instead of using one phase merely as an inert support.

Despite promising laboratory results, application-oriented investigations face common practical constraints. First, most high-performance data originate from high-intensity ultrasonic tests in batch reactors, while material behaviors under real-world low-magnitude irregular mechanical stimuli (ocean waves, fluid turbulence, industrial vibration) are insufficiently explored. Second, methodological ambiguities persist. Acoustic cavitation, thermochemical heating and sonoluminescence under ultrasound create non-negligible side reactions. Many reports lack complete standardized controls to distinguish authentic piezocatalysis from sonochemical artifacts, hampering cross-study performance comparison and reliable structure–activity correlation construction. Third, unavoidable material trade-offs exist: zwitterionic COFs achieve strong ionic-displacement polarization for water splitting at the cost of reduced crystallinity; high-activity heterostructures require robust interfacial coupling, and weak interfaces degrade strain transmission and charge transport. For COF-based PENGs, acceptable open-circuit voltages have been achieved, yet low output-current density limits real-world self-powered electronics deployment.

## 5. Existing Challenges and Future Perspectives

Despite considerable progress, COFs-based piezoelectric materials are still in the infant research stage, with multiple bottlenecks remaining to be addressed before practical deployment.

Piezoelectric coefficient limitation: Compared with traditional inorganic ferroelectrics, most COFs still possess relatively low intrinsic piezoelectric response. A core challenge lies in the inverse correlation between molecular dipole strength and macroscopic polarization efficiency: monomers that generate strong molecular dipoles typically introduce large steric hindrance that disrupts long-range crystallographic order, which in turn reduces dipole alignment and weakens net macroscopic polarization. Rational molecular-level design is required to further enhance intrinsic lattice polarization while maintaining sufficient crystallinity. Recent progress demonstrates that constructing zwitterionic skeletons to introduce large Born-effective-charge-governed ionic-displacement polarization is a promising route to lift piezoelectric performance of organic porous frameworks. At present, systematic structure-piezoelectric-property database for COFs is absent. How to balance crystallinity, porosity and non-centrosymmetric dipole density is a core material-design puzzle; for ionic/zwitterionic COFs, the trade-off between strong ionic polarization and Coulomb-repulsion-caused crystallinity degradation deserves special attention.

Mechanistic ambiguity: The correlation between flexible linker torsion, pore deformation, and piezoresponse lacks quantitative theoretical models. The modern theory of polarization (Berry-phase approach) combined with density-functional-perturbation-theory-calculated Born effective charges offers a powerful tool to quantify ionic-displacement- versus electronic-distortion-originated polarization for COFs systems, yet relevant quantitative calculations are still scarce for most COFs piezocatalysts. Systematic operando characterizations under low-frequency, weak-vibration conditions are insufficient. Currently, most piezocatalytic performance evaluations rely on ultrasonic excitation, yet standardized control protocols to distinguish intrinsic piezoelectric activity from sonochemical side effects remain far from universally adopted. For high-porosity COFs systems, enhanced interfacial mass transfer may further amplify the contribution of acoustic cavitation effects, leading to potential overestimation of piezocatalytic performance. The lack of rigorous control experiments not only affects the accuracy of structure–performance relationship analysis, but also hinders fair comparison across different material systems. Future research should establish standardized control experimental procedures as a community standard, including isothermal reaction control, non-piezoelectric material reference, and excitation parameter-dependent activity analysis. Alternative mechanical excitation modes such as cyclic compression, fluid shear and mechanical vibration that introduce minimal thermal effects are also recommended to validate the intrinsic piezoelectric contribution.

As elaborated in Section 2.1.1, PFM measurements on soft porous COFs suffer multiple measurement artifacts. Standardized PFM protocols together with cross-validation by second-harmonic generation (SHG), transient piezoelectric current and bulk piezoelectric tests are strongly recommended.

Very recently, quantitative experimental protocols to distinguish intrinsic lattice piezoelectricity from non-piezoelectric side-reactions have been demonstrated for piezocatalytic systems [82]. Similar rigorous experimental workflows are urgently required for COFs-based catalysts. This work highlights that merely observing enhanced catalytic activity under mechanical excitation cannot serve as sufficient proof of intrinsic piezocatalysis. Well-designed control experiments combining multiple characterization tools including second-harmonic generation, transient piezoelectric current measurement and varied-mode excitation tests are mandatory to correlate real piezoelectric polarization with measured catalytic performance.

Synthesis and processability obstacles: High-quality single-crystal piezoelectric COFs synthesis remains difficult. Most reported COF piezocatalysts are powder samples. Fabrication of large-area, stable, self-standing films and monolithic devices is challenging. Interfacial compatibility issues emerge when compositing COFs with polymer substrates. Powder COFs materials are difficult to assemble directly into practical devices; powder recycling and separation in aqueous catalytic systems also bring engineering difficulties. For zwitterionic COFs, synthetic routes to improve crystallinity while preserving zwitterionic charge characteristics need further development. For heterostructure systems, controllable construction of intimate covalent or supramolecular interfacial contact rather than simple physical mixing remains a key synthetic challenge.

Practical-environment stability: Long-term performance stability under humidity, temperature fluctuations, and complex actual water matrices needs further verification. Although COFs’ covalent linkages exhibit superior chemical resistance, mechanical fatigue under long-term cyclic strain may induce microcracks and partial amorphization of COFs, leading to a gradual attenuation of piezoresponse. Related fatigue behavior investigations are very limited so far. For zwitterionic COFs, the structural stability of ionic moieties under long-term cyclic mechanical stress and aqueous redox conditions requires more experimental validation. For composite catalysts, interfacial debonding between COF and inorganic/polymer phases under cyclic mechanical loading is another non-negligible failure mode.

Device-level engineering gaps: For piezoelectric energy harvesting applications, current research is mostly at the proof-of-concept stage, and device-level engineering remains a major gap. The lack of standardized device fabrication and testing protocols leads to large variations in reported performance data, making cross-study quantitative comparison difficult. Key device-level factors including active layer thickness control, electrode interface optimization, packaging damping reduction, and force transmission efficiency have not been systematically investigated for COFs-based systems.

Future research should establish standardized device fabrication and testing protocols for COFs-based PENGs, with unified excitation parameters, device dimensions and electrode configurations. Systematic device-level optimization should be carried out to improve output power density and long-term stability to promote the transition from material-level property verification to practical device applications.

Practical deployment barriers: Beyond material-level performance optimization, multiple engineering barriers remain to be addressed before practical deployment. First, the excitation condition mismatch between laboratory tests and real-world scenarios is prominent. Most high-performance piezocatalytic results are obtained under high-power, high-frequency ultrasonic excitation, which is far from the low-frequency, low-amplitude, irregular mechanical energy available in natural environments such as ocean waves, water flow and industrial vibration. The performance of COFs piezocatalysts under realistic weak mechanical excitation remains largely uncharacterized, and frequency-matching structural design is needed to efficiently harvest low-grade mechanical energy.

Second, device-level engineering challenges constrain practical application. Powder-form COFs catalysts are difficult to recover and reuse in continuous-flow treatment systems, and immobilization often leads to reduced mass transfer efficiency and blocked active sites. For piezoelectric energy harvesting devices, the relatively low output current density of COFs-based nanogenerators limits their ability to drive practical electronic loads. Issues including electrode contact stability, flexible packaging encapsulation and long-term environmental durability also need to be addressed for device-level deployment.

Third, scalable and cost-effective manufacturing of piezoelectric COFs remains challenging. Current lab-scale solvothermal synthesis requires long reaction times, high temperature and large amounts of organic solvents, with low batch yield and poor consistency. The development of continuous, microwave-assisted or mechanochemical synthesis routes is necessary for large-scale production. For thin-film and device applications, large-area, uniform and reproducible fabrication of piezoelectric COFs films remains technically demanding.

Promising future research directions: Develop high-quality single-crystal piezoelectric COFs, combine first-principles polarization calculation and molecular dynamic (MD) simulation to quantitatively disclose structure–polarization relationships. Single-crystal samples eliminate grain-boundary interference, which enables accurate measurement of intrinsic piezoelectric parameters and deep mechanistic understanding. Specifically, applying modern theory of polarization and Born-effective-charge analysis to decompose ionic-displacement and electronic-distortion polarization contributions will deepen atomic-level comprehension for ionic/zwitterionic COFs piezocatalysts.

Develop multi-modal operando characterization techniques tailored for piezocatalytic systems. Develop in situ liquid-cell PFM, time-resolved Kelvin probe force microscopy (KPFM) under periodic strain, and in situ SHG imaging to capture real-time evolution of piezopotential and local polarization under catalytic working conditions. Combine electrochemistry-mechanical coupling measurements, in situ electron paramagnetic resonance (EPR) and in situ Fourier-transform infrared spectroscopy (FTIR) to track charge-transport dynamics and reactive intermediate evolution. Establish standardized control protocols to distinguish true piezocatalytic activity from sonochemical and other side effects, unify performance-testing conditions including mechanical excitation mode, power, frequency, reaction temperature, to realize fair performance comparison across different literature reports.

Explore novel performance regulation strategies beyond existing modification paradigms. Develop topological symmetry engineering, interlayer van der Waals polarization modulation, framework flexibility strain engineering, and confined local dipole engineering to further boost intrinsic piezoresponse. Exploit high-throughput screening and machine-learning workflows as auxiliary tools to mine non-centrosymmetric COFs candidates with outstanding piezoresponse, especially zwitterionic COFs candidates with large Born effective charges and mitigated Coulomb-repulsion-driven crystallinity loss. Construct material database linking monomer geometry, space group, skeleton flexibility and piezoelectric coefficient, accelerating experimental exploration cycle.

Optimize scalable synthesis and device fabrication strategies. Develop microwave-assisted continuous synthesis and interface-induced oriented growth methods to achieve large-scale production of highly crystalline piezoelectric COFs. Fabricate large-area, stable self-standing films and monolithic devices, and optimize interfacial compatibility between COFs and polymer substrates. Prefer covalent or supramolecular tight interfacial contact instead of simple physical blending for heterostructure systems to ensure efficient strain transfer and charge coupling.

Expand application boundaries toward high-value practical scenarios. Beyond conventional energy conversion and environmental remediation, extend piezoelectric COFs applications to ocean wave energy harvesting coupled with uranium resource extraction, industrial low-frequency residual vibration recovery, in situ groundwater remediation, piezoelectric sonodynamic therapy and pathogen-selective microbiome regulation for biomedical use. Combine the biocompatibility and porous characteristics of organic COFs with piezoelectric effect to open interdisciplinary research branches.

Establish multi-scale theoretical frameworks for mechanism interpretation. Beyond phenomenological description, develop quantitative multi-scale models that bridge molecular-level dipole generation, lattice-level polarization propagation, interfacial charge transfer and surface catalytic reaction kinetics. Clarify the coupling relationship among molecular symmetry, dipole orientation, crystallinity, porosity, conductivity, charge transport, and mechanical deformation, and establish quantitative structure–activity relationships to guide rational material design.

To address practical deployment barriers, future research should prioritize robust linkage engineering to enhance chemical stability of piezoelectric COFs under harsh aqueous and redox conditions. Interfacial reinforcement strategies, including covalent bonding and supramolecular crosslinking, should be developed to improve mechanical fatigue resistance of heterostructure and composite systems. Anti-fouling surface modification and anti-interference structure design are needed to adapt to complex real water matrices. For practical energy harvesting, frequency-tunable structural design should be explored to match the frequency range of natural low-grade mechanical energy sources. Scalable continuous synthesis protocols and device integration technologies should be developed to promote the transition from lab-scale demonstrations to practical applications.

## 6. Conclusions

Piezoelectric-related covalent-organic-frameworks (COFs)-based systems represent an emerging class of porous crystalline materials merging molecular tunability, permanent porosity and mechanical–electrical coupling capability. This review categorizes these systems into four major groups: intrinsic piezoelectric COFs, COFs–polymer composites, single-piezo-phase heterostructures, and dual-piezoelectric heterostructures. A core conceptual advance of this work is explicitly differentiating intrinsic lattice-dipole polarization originating from COFs skeletons from extrinsic contributions generated by external inorganic piezoelectric phases, heterojunction interfaces or polymer matrices. For composite and heterostructure systems, improved catalytic or energy-harvesting performance cannot automatically be ascribed to the intrinsic piezoelectric response of the COFs component alone, which helps avoid misattributing functional improvements purely to COFs-inherent piezoelectricity.

Three dominant theoretical frameworks (energy-band theory, screening-charge-effect theory, displacement-current theory) are widely invoked to interpret piezocatalytic and piezo-photocatalytic behaviors. Nevertheless, definitive operando experimental evidence to validate these competing hypotheses remains lacking. Most existing understandings are built on computational simulations and indirect experimental observations. Measurement pitfalls including PFM artifacts and sonochemical side effects from ultrasonic excitation frequently confound experimental outputs, indicating enhanced activity under mechanical stimulation cannot be simply ascribed to piezoelectric polarization.

Five multi-scale modification approaches can improve COFs piezoresponse and catalytic performance, yet each strategy carries inherent structural trade-offs. Maximizing molecular-dipole strength may introduce severe steric hindrance and degrade long-range crystallinity. Over-generated defects disrupt dipole ordering and weaken macroscopic polarization. Material optimization therefore demands balanced tuning among symmetry breaking, dipole ordering, crystallinity, porosity and charge-transport properties rather than maximizing any single parameter. For heterostructures, interfacial contact quality is decisive: robust covalent or supramolecular interfaces guarantee efficient strain and charge transfer, while simple physical mixing yields poor piezo-coupling effects.

Piezoelectric COFs exhibit promising lab-scale performance in water-energy conversion, CO_2_ photoreduction, pollutant remediation, heavy-metal removal, antibacterial therapy and piezoelectric nanogenerators. However, most catalytic metrics are collected under high-power ultrasonic batch conditions, and experimental set-ups vary widely across publications. Direct cross-paper numerical comparison should be treated with caution, highlighting the urgent need for community-accepted standardized testing benchmarks. Although COFs-based PENG prototypes achieve competitive open-circuit voltage, low output-current density remains a key practical bottleneck compared with conventional inorganic piezoceramics.

In summary, this field still faces prominent bottlenecks including unresolved mechanistic controversies, measurement uncertainties, synthetic challenges for high-crystallinity intrinsic piezoelectric COFs, insufficient long-term stability data under realistic complex media, and large performance gaps between ultrasonic-lab conditions and natural low-grade mechanical excitations. Establishing standardized characterization protocols and bridging material-level lab demonstrations toward practical device implementation constitute the most critical next steps for piezoelectric COFs research.

## Figures and Tables

**Table 1:** Summary of structural design and modification strategies for intrinsic piezoelectric COFs and COFs-containing composite/hybrid systems and their representative applications.

Modification Strategy	Subcategory/Typical Systems	Core Mechanism	Applications	Experimental Conditions	Mechanical Stress Source	Catalytic Performance/Piezoelectric Performance	Reference
Building-block molecular design	Twisted units (Cz-COFs, COF-DH-Eth), intrinsic COFs	Non-planar conformation breaks centrosymmetry to form intrinsic molecular dipoles	Piezo-photocatalytic H_2_O_2_ production	50 mg catalyst dispersed in 25 mL water	50 W, 40 kHz, 300 W Xe lamp with a UV cut-off filter (>420 nm)	9212 μmol g^−1^ h^−1^	[52]
	COF/polymer systems, polar-group/fluorinated-chain (CityU-13)	Polar bonds generate oriented local dipoles for macroscopic piezoelectricity	Piezoelectric nanogenerators	Al/CityU-13/Al device; quasi-static compressive test for solid-state energy-harvesting evaluation	External cyclic compressive force (quasi-static, unspecified magnitude)	60 V	[53]
	Propeller-like units (TAPA-PDA COF), intrinsic COFs	Steric hindrance induces permanent molecular dipoles	Piezocatalytic water-splitting H_2_ production	5 mg catalyst dispersed in 30 mL water	40 kHz, 200 W	6.6 mmol g^−1^ h^−1^	[54]
	Donor–acceptor (D–A) skeletons (TP-BT-0F-COF series), intrinsic COFs	D–A interaction creates in-plane dipoles; deformation amplifies polarization	Piezo-photocatalytic water splitting; co-generation of H_2_/H_2_O_2_	2 mg catalyst dispersed in 20 mL water	60 W, 40 kHz, 300 W Xe lamp with a UV cut-off filter (>420 nm)	1501.4 (H_2_) and 1435.8 (H_2_O_2_) μmol g^−1^ h^−1^	[55]
	Zwitterionic building blocks (SQ-TAPT), intrinsic COFs	Large Born effective charges enable strong ionic-displacement polarization	High co-catalyst-free piezocatalytic H_2_ evolution	10 mg catalyst dispersed in 180 mL water and 20 mL CH_3_OH	75 kHz, 300 W	745.9 μmol g^−1^ h^−1^	[56]
Defect engineering	Missing-linker vacancies (D-COF@ZnO), intrinsic COFs	Local lattice distortion breaks inversion symmetry to form localized polarization domains	U(VI) removal	5 mg of catalyst dispersed in 100 mg L^−1^ of 20 mL U(VI) solution	40 kHz, 120 W	2010.65 mg g^−1^	[57]
Morphology modulation	1D tubular CTN-1, intrinsic COFs	Tubular geometry concentrates mechanical stress for large dipole oscillation	Piezo-catalytic H_2_O_2_ production	2 mg catalyst dispersed in 10 mL water	40 kHz, 100 W	4115 μmol g^−1^ h^−1^	[32]
Single-atom metal anchoring	Sb-SASb-TpBpy-COF, intrinsic COFs	Coordination-induced local lattice distortion generates extra dipoles	Piezo-catalytic H_2_O_2_ production	5 mg catalyst dispersed in 20 mL water	40 kHz, 110 W	1500.58 µmol g^−1^ h^−1^	[58]
	Zn-Por-COF, intrinsic COFs	Single-atom sites construct intrinsic piezo-internal field	piezo-photocatalytic CO_2_-to-CO conversion	10 mg catalyst, 10 mg [Ru(bpy)_3_]Cl_2_·6H_2_O, 10 mg 1,3-Dimethyl-2-phenylbenzimidazoline dispersed in 8 mL acetonitrile and 2 mL H_2_O	100 W, 40 kHz, 300 W Xe lamp with a UV cut-off filter (>420 nm)	15.59 mmol g^−1^ h^−1^	[59]
	Dual-single-atom systems (Sb-Fe-COF), intrinsic COFs	Dual atomic sites for polarization promotion and independent catalysis	piezocatalytic advanced oxidation	5 mg of catalyst dispersed in 10 mg L^−1^ of 25 mL SMX solution	40 kHz, 110 W	88.34% within 60 min	[60]
Heterostructure construction	BTW-40@TpPa, single-piezoelectric-phase	Synergy of piezopotential and optimized interfacial band-alignment	Piezo-photocatalytic H_2_ evolution	5 mg catalyst, 0.1 mol L^−1^ ascorbic acid aqueous solution (160 mL) and 1 g L^−1^ H_2_PtCl_6_ (66 µL) as a cocatalyst	300 W, 45 kHz, 300 W Xe lamp with a UV cut-off filter (>420 nm)	33 mmol g^−1^ h^−1^	[61]
	BiFeO_3_@TpPa-1-COF, single-piezoelectric-phase	Synergy of piezopotential and optimized interfacial band-alignment	Piezo-photocatalytic H_2_ and O_2_ evolution	10 mg catalyst dispersed in 10 mL H_2_O	100 W, 40 kHz, 300 W Xe lamp with a UV cut-off filter (>420 nm)	1416.4 (H_2_) and 708.2 (O_2_) μmol h^−1^ g^−1^	[62]
	TpDb-COF/BTO, single-piezoelectric-phase	Z-scheme + covalent linkage; ferroelectric polarization synergy	Piezo-photocatalytic H_2_ evolution	5 mg catalyst, 100 mg ascorbic acid (50 mL) and 3wt% Pt (20 µL) as a cocatalyst	300 W, 300 W Xe lamp with a UV cut-off filter (>420 nm)	10.4 mmol g^−1^ h^−1^	[63]
	BTO@TD-COF, single-piezoelectric-phase	Core–shell; surface enrichment; polarization-driven charge separation	Adsorption-enhanced BPA degradation	5 mg of catalyst dispersed in 20 mg L^−1^ of 50 mL BPA solution	180 W, 40 kHz, 200 W Xe lamp with a UV cut-off filter (>420 nm)	100% within 75 min	[64]
	PTO@COF, single-piezoelectric-phase	Covalently bonded perovskite-imine-COF; mechanochemical synthesis	Wastewater remediation	10 mg of catalyst dispersed in 20 mg L^−1^ of 50 mL MB solution	40 kHz, 140 W	100% within 40 min	[65]
	BTO@β-COF, single-piezoelectric-phase	Confined-space micro-reactor; piezo-self-Fenton; ~100% ·OH yield	Antibiotic degradation	50 mg of catalyst dispersed in 10 mg L^−1^ of 100 mL SMX solution	40 kHz, 480 W	96.1% within 120 min	[66]
	MOC-Q3/EA-COF, dual-piezoelectric-phase	Direct Z-scheme MOC/COF	Overall water splitting	2 mg catalyst dispersed in 20 mL H_2_O	60 W,40 kHz, 300 W Xe lamp with a UV cut-off filter (>420 nm)	293.94 (H_2_) and 400.38 (H_2_O_2_) μmol h^−1^ g^−1^	[67]
	MOC-FA3/S-COF, dual-piezoelectric-phase	Dual-piezoelectric Z-scheme; both subunits intrinsic piezo	Piezo-photocatalytic H_2_/H_2_O_2_ co-production	2 mg catalyst dispersed in 20 mL H_2_O	60 W,40 kHz, 300 W Xe lamp with a UV cut-off filter (>420 nm)	334.3 (H_2_) and 203.6 (H_2_O_2_) μmol h^−1^ g^−1^	[68]
	Non-ferroelectric semiconductor@COF (ZnO@COF), single-piezoelectric-phase	Z-scheme interface amplifies strain-driven polarization	Wave-driven piezocatalytic uranium extraction from seawater	5 mg of catalyst dispersed in 100 mg L^−1^ of 15 mL U(VI) solution	120 W, 40 kHz,	7.56 mg g^−1^d^−1^	[69]
	COF/polymer systems, (COF/PVDF-HFP)	COF optimizes polymer electroactive phase; forms (step scheme) S-scheme heterojunction	Flexible device for degradation and antibacterial applications	0.1 g of the composite films immersed in 10 mL of RhB aqueous solution (20 mg L^−1^)	100 W, 40 kHz, 15 A lamp with the incident light intensity (100 mW cm^−2^)	97.73% within 180 min	[70]
	COF/polymer systems, BaBi_4_Ti_4_O_15_@COFs/PVDF-TrFE	Aurivillius ferroelectric + COF; enhanced built-in field; flexible membrane	Piezo-photocatalytic H_2_ production	10 membranes about 0.6 cm^2^ in size were directly immersed in 100 mL of solution	150 W, 40 kHz, 300 W Xe lamp simulating full-spectrum solar light	2072.3 µmol h^−1^ m^−2^	[71]
	COF/polymer systems, NiFe-LDH/MXene/COF-LZU1	Ternary heterostructure; dual-pathway; MXene mediator	H_2_O_2_ synthesis + dye degradation	50 mg of the catalyst dispersed in 100 mL of deionized water, 50 mg of catalyst dispersed in 10 mg L^−1^ of 100 mL MB solution	300 W, 40 kHz	2966.223 μmol L^−1^ in 7 h, 98.4% within 3.5 h	[72]
	Cu-modified ZnO/P-COF, single-piezoelectric-phase	S-scheme; internal electric field + polarized field synergy	Piezo-photocatalytic H_2_O_2_ production	30 mg catalyst dispersed in 60 mL H_2_O	200 W, 40 kHz, 300 W Xe lamp with a UV cut-off filter (350 nm–780 nm)	1838.8 μmol g^−1^ h^−1^	[73]
	Ag@Ph-g-C_3_N_4_/P-COF, single-piezoelectric-phase	Lewis–Brønsted acids; D–A_1_–A_2_; Pauling-type O_2_ adsorption	Piezo-photocatalytic H_2_O_2_ production	50 mg catalyst dispersed in 90 mL water and 10 mL IPA	50 W, 40 kHz, 300 W Halogen lamp with a UV cut-off filter (>420 nm)	1018.63 ± 50.93 μmol h^−1^	[74]
	ZnO@Viologen-COF, single-piezoelectric-phase	Redox-switchable; pathogen-triggered lattice distortion; piezo-phototropic	Pathogen-selective microbiome regulation	25 mg of catalyst dispersed in 10 mg L^−1^ of 50 mL MB solution	1 W cm^−2^, 1.0 MHz, 50% duty cycle	83% within 10 min	[75]
	COF/polymer systems, Por-COF	COF–piezoelectric aerogel hybrid; solar-mechanical coupling	Seawater desalination via interfacial evaporation	150 mg Por-COFs spray-coated onto poled PVDF-TrFE aerogel (corona poling 17 kV); 1-sun illumination; brine/dye solutions; outdoor test 9:00–17:00	Low-frequency wave energy (1 Hz) simulated by a 20 W water pump (13,000 L h^−1^)	2.82 ± 0.3 kg m^−2^ h^−1^	[76]

Note: For composite systems combining COFs with external inorganic piezoelectric phases or polymer matrices, observed performance enhancements are not automatically assignable to intrinsic piezoelectricity originating from the COFs lattice. Contributions may stem from the inorganic piezoelectric component, polymer matrix, heterojunction interfacial electric-field effects or interfacial strain-transfer effects.

**Table 2:** Summary of representative applications, material systems and performance remarks for intrinsic piezoelectric COFs and COF-containing composite/hybrid piezo-photocatalysis and energy-harvesting devices.

Application Category	Typical Material Systems	Experimental Conditions	Mechanical Stress Source	Main Performance and Remarks	Reference
Water conversion (H_2_ production)	SQ-TAPT (zwitterionic COF), intrinsic COFs	10 mg catalyst dispersed in 180 mL water and 20 mL CH_3_OH	75 kHz, 300 W	745.9 μmol g^−1^ h^−1^	[56]
Water conversion (H_2_ production)	TAPA-PDA, intrinsic COFs	5 mg catalyst dispersed in 30 mL H_2_O	40 kHz, 200 W	6.6 mmol g^−1^ h^−1^	[54]
Water conversion (H_2_ production)	COF-TpDb@BTO, single-piezoelectric-phase	5 mg catalyst, 100 mg ascorbic acid (50 mL) and 3 wt% Pt (20 µL) as a cocatalyst	300 W, 300 W Xe lamp with a UV cut-off filter (>420 nm)	10.4 mmol g^−1^ h^−1^	[63]
Water conversion (H_2_O_2_ production)	COF-DH-Eth, intrinsic COFs	50 mg catalyst dispersed in 25 mL H_2_O	50 W, 40 kHz, 300 W Xe lamp with a UV cut-off filter (>420 nm)	9212 μmol g^−1^ h^−1^	[52]
Water conversion (H_2_/H_2_O_2_ production)	MOC-FA3/S-COF, dual-piezoelectric-phase	2 mg catalyst dispersed in 20 mL H_2_O	60 W, 40 kHz, 300 W Xe lamp with a UV cut-off filter (>420 nm)	334.3 (H_2_) and 203.6 (H_2_O_2_) μmol h^−1^ g^−1^	[68]
Water conversion (H_2_O_2_ production)	BaTiO_3_@COF, single-piezoelectric-phase	50 mg catalyst dispersed in 100 mL H_2_O	300 W	240.7 µmol g^−1^ h^−1^	[77]
Water conversion (H_2_O_2_ production)	COF/polymer systems, NiFe-LDH/MXene/COF-LZU1	50 mg of the catalyst dispersed in 100 mL of H_2_O	300 W, 40 kHz	423.74 μmol L^−1^ h^−1^	[72]
	CTN-1 (1D covalent triazine nanotube), intrinsic COFs	2 mg catalyst dispersed in 10 mL H_2_O	40 kHz, 100 W	4115 μmol g^−1^ h^−1^	[32]
Piezo-photocatalytic CO_2_ reduction	Zn-Por-COF, intrinsic COFs	10 mg catalyst, 10 mg [Ru(bpy)_3_]Cl_2_·6H_2_O, 10 mg 1,3-Dimethyl-2-phenylbenzimidazoline dispersed in 8 mL acetonitrile and 2 mL H_2_O	100 W, 40 kHz, 300 W Xe lamp with a UV cut-off filter (>420 nm)	15.59 mmol g^−1^ h^−1^	[59]
Organic-pollutant degradation	PTO@COF, single-piezoelectric-phase	10 mg of catalyst dispersed in 20 mg L^−1^ of 50 mL MB solution	40 kHz, 140 W	100% within 40 min	[65]
	Dual-single-atom systems (Sb-Fe-COF), intrinsic COFs	5 mg of catalyst dispersed in 10 mg L^−1^ of 25 mL SMX solution	40 kHz, 110 W	88.34% within 60 min	[60]
	BTO@TD-COF, single-piezoelectric-phase	5 mg of catalyst dispersed in 20 mg L^−1^ of 50 mL BPA solution	180 W, 40 kHz, 200 W Xe lamp with a UV cut-off filter (>420 nm)	100% within 75 min	[64]
	Bi_2_WO_6_/R-COFs, single-piezoelectric-phase	5 mg of catalyst dispersed in 20 mL SMX solution of 5 ppm	40 W, 100 kHz, 200 W Xe lamp with a UV cut-off filter (>420 nm)	93.5% within 48 min	[78]
Antibacterial disinfection	ZnO@Viologen-COF (ZVP), single-piezoelectric-phase	Bacteria initial concentration: 2 × 10^7^ CFU mL^−1^ Catalyst concentration (ZVP): 300 μg mL^−1^ in saline	80 W LED + ultrasound (1 W cm^−2^, 1.0 MHz, 50% duty-cycle	99.5% within 10 min	[75]
	COF/polymer systems, COF/PVDF-HFP	Gram-negative *E. coli* and Gram-positive *S. aureus* served as model microbes. Composite films were immersed in sterile-growth-medium-based bacterial suspensions (10^6^ CFU mL^−1^).	100 W, 40 kHz, 15 A Xe lamp with the incident light intensity (100 mW cm^−2^)	99% within 30 min	[70]
Piezocatalytic U(VI) reduction	D-COF@ZnO, single-piezoelectric-phase	5 mg of catalyst dispersed in 100 mg L^−1^ of 20 mL U(VI) solution	40 kHz, 120 W	2010.65 mg g^−1^	[57]
	ZnO@COF, single-piezoelectric-phase	5 mg of catalyst dispersed in 100 mg L^−1^ of 15 mL U(VI) solution	120 W, 40 kHz,	7.56 mg g^−1^ d^−1^	[69]
Piezoelectric nanogenerators (PENGs, mechanical-energy harvesting)	COF-DH-Eth (pristine COF-based PENG device)	solid-state powder-based device	4 Hz cyclic compression (20 N)	6 V	[52]
	COF-DH-H (pristine COF-based PENG device)	solid-state powder-based device	4 Hz cyclic compression (20 N)	4 V	[52]
	CityU-14 (pristine COF-based PENG device)	Sandwiched Al/CityU-14/Al device; quasi-static compressive test for solid-state energy-harvesting evaluation	External cyclic compressive force (quasi-static, unspecified magnitude)	50 V	[53]
	CityU-13 (pristine COF-based PENG device)	Sandwiched Al/CityU-13/Al device; quasi-static compressive test for solid-state energy-harvesting evaluation	External cyclic compressive force (quasi-static, unspecified magnitude)	60 V	[53]

Note: For COF-inorganic and COF–polymer composite entries, measured activity or electrical output cannot be simplistically attributed to intrinsic piezoelectric polarization of the COF framework. Additional contributions from inorganic piezoelectric phases, polymer matrices or heterojunction effects should be considered.

## Data Availability

No new data were created or analyzed in this study. Data sharing is not applicable to this article.

## Abbreviations

The following abbreviations are used in this manuscript.

COFs	covalent organic frameworks
MOFs	metal–organic frameworks
PENG	piezoelectric nanogenerator
PFM	piezoresponse force microscopy
BET	Brunauer–Emmett–Teller
D–A	Donor–acceptor
CTN-1	covalent triazine nanotube 1
DFT	density functional theory
SQ-TAPT	squaric-acid-based zwitterionic TAPT covalent organic framework
PDA-TAPT	terephthaldehyde and 4,4′,4″-(1,3,5-triazine-2,4,6-triyl)trianiline-based covalent organic framework
TpPa-1	1,3,5-triformylphloroglucinol-p-phenylenediamine-based covalent organic framework
S-scheme	step scheme
SHG	second-harmonic generation
MD	molecular dynamics
KPFM	Kelvin probe force microscopy
EPR	electron paramagnetic resonance
FTIR	Fourier-transform infrared spectroscopy
